# Spatial Transcriptomics of Human Decidua Identifies Molecular Signatures in Recurrent Pregnancy Loss

**DOI:** 10.1093/gpbjnl/qzaf080

**Published:** 2025-10-01

**Authors:** Qing Sha, Qiaoni Yu, Kaixing Chen, Junyu Wang, Feiyang Wang, Chen Jiang, Yuanzhe Li, Meifang Tang, Yanbing Hou, Ke Liu, Kun Chen, Zongcheng Yang, Shouzhen Li, Jingwen Fang, Sihui Luo, Xueying Zheng, Jianping Weng, Kun Qu, Chuang Guo

**Affiliations:** Department of Oncology, The First Affiliated Hospital of USTC, State Kye Laboratory of Eye Health, School of Basic Medical Sciences, Division of Life Sciences and Medicine, University of Science and Technology of China, Hefei 230021, China; Department of Oncology, The First Affiliated Hospital of USTC, State Kye Laboratory of Eye Health, School of Basic Medical Sciences, Division of Life Sciences and Medicine, University of Science and Technology of China, Hefei 230021, China; Department of Oncology, The First Affiliated Hospital of USTC, State Kye Laboratory of Eye Health, School of Basic Medical Sciences, Division of Life Sciences and Medicine, University of Science and Technology of China, Hefei 230021, China; Institute of Artificial Intelligence, Hefei Comprehensive National Science Center, Hefei 230088, China; Department of Oncology, The First Affiliated Hospital of USTC, State Kye Laboratory of Eye Health, School of Basic Medical Sciences, Division of Life Sciences and Medicine, University of Science and Technology of China, Hefei 230021, China; State Key Laboratory of Stem Cell and Reproductive Biology, Key Laboratory of Organ Regeneration and Reconstruction, Institute of Zoology, Chinese Academy of Sciences, Beijing 100101, China; Department of Oncology, The First Affiliated Hospital of USTC, State Kye Laboratory of Eye Health, School of Basic Medical Sciences, Division of Life Sciences and Medicine, University of Science and Technology of China, Hefei 230021, China; Institute of Artificial Intelligence, Hefei Comprehensive National Science Center, Hefei 230088, China; Department of Oncology, The First Affiliated Hospital of USTC, State Kye Laboratory of Eye Health, School of Basic Medical Sciences, Division of Life Sciences and Medicine, University of Science and Technology of China, Hefei 230021, China; Institute of Artificial Intelligence, Hefei Comprehensive National Science Center, Hefei 230088, China; Department of Oncology, The First Affiliated Hospital of USTC, State Kye Laboratory of Eye Health, School of Basic Medical Sciences, Division of Life Sciences and Medicine, University of Science and Technology of China, Hefei 230021, China; Department of Oncology, The First Affiliated Hospital of USTC, State Kye Laboratory of Eye Health, School of Basic Medical Sciences, Division of Life Sciences and Medicine, University of Science and Technology of China, Hefei 230021, China; Department of Oncology, The First Affiliated Hospital of USTC, State Kye Laboratory of Eye Health, School of Basic Medical Sciences, Division of Life Sciences and Medicine, University of Science and Technology of China, Hefei 230021, China; Department of Oncology, The First Affiliated Hospital of USTC, State Kye Laboratory of Eye Health, School of Basic Medical Sciences, Division of Life Sciences and Medicine, University of Science and Technology of China, Hefei 230021, China; Department of Oncology, The First Affiliated Hospital of USTC, State Kye Laboratory of Eye Health, School of Basic Medical Sciences, Division of Life Sciences and Medicine, University of Science and Technology of China, Hefei 230021, China; Department of Stomatology, The First Affiliated Hospital of USTC, Division of Life Sciences and Medicine, University of Science and Technology of China, Hefei 230021, China; Department of Oncology, The First Affiliated Hospital of USTC, State Kye Laboratory of Eye Health, School of Basic Medical Sciences, Division of Life Sciences and Medicine, University of Science and Technology of China, Hefei 230021, China; Department of Oncology, The First Affiliated Hospital of USTC, State Kye Laboratory of Eye Health, School of Basic Medical Sciences, Division of Life Sciences and Medicine, University of Science and Technology of China, Hefei 230021, China; HanGene Biotech, Xiaoshan Innovation Polis, Hangzhou 311200, China; Department of Endocrinology, Institute of Endocrine and Metabolic Diseases, The First Affiliated Hospital of USTC, Division of Life Sciences and Medicine, Clinical Research Hospital of Chinese Academy of Sciences (Hefei), University of Science and Technology of China, Hefei 230001, China; Department of Endocrinology, Institute of Endocrine and Metabolic Diseases, The First Affiliated Hospital of USTC, Division of Life Sciences and Medicine, Clinical Research Hospital of Chinese Academy of Sciences (Hefei), University of Science and Technology of China, Hefei 230001, China; Department of Endocrinology, Institute of Endocrine and Metabolic Diseases, The First Affiliated Hospital of USTC, Division of Life Sciences and Medicine, Clinical Research Hospital of Chinese Academy of Sciences (Hefei), University of Science and Technology of China, Hefei 230001, China; Department of Oncology, The First Affiliated Hospital of USTC, State Kye Laboratory of Eye Health, School of Basic Medical Sciences, Division of Life Sciences and Medicine, University of Science and Technology of China, Hefei 230021, China; Institute of Artificial Intelligence, Hefei Comprehensive National Science Center, Hefei 230088, China; School of Biomedical Engineering, Suzhou Institute for Advanced Research, University of Science and Technology of China, Suzhou 215123, China; Department of Oncology, The First Affiliated Hospital of USTC, State Kye Laboratory of Eye Health, School of Basic Medical Sciences, Division of Life Sciences and Medicine, University of Science and Technology of China, Hefei 230021, China; Department of Rheumatology and Immunology, The First Affiliated Hospital of USTC, Division of Life Sciences and Medicine, University of Science and Technology of China, Hefei 230021, China; School of Pharmacy, Bengbu Medical University, Bengbu 233030, China

**Keywords:** Spatial transcriptomic analysis, Early pregnancy, Decidual natural killer cell, Transcriptional regulation, Immune toleranc, e

## Abstract

The human decidua establishes immune tolerance at the maternal–fetal interface and is essential for successful embryo implantation and development. Here, we conducted a spatial transcriptomic analysis of human decidua from early pregnancies in both healthy donors and patients with recurrent pregnancy loss (RPL). Our analysis revealed two distinct spatial domains, named implantation zone (IZ) and glandular-secretory zone (GZ), corresponding to the layers of decidua compacta and spongiosa, respectively. The decidual natural killer cell subset (dNK1) and the decidual macrophage subset (dM2), both associated with growth promotion and immune regulation, were predominantly localized in the healthy IZ but were significantly reduced in RPL patients. In contrast, cytotoxic CD8^+^ T cells, sparsely distributed in the healthy decidual IZ and GZ domains, were elevated in both domains under RPL conditions. Spatial cell–cell interaction analysis indicated a broad exhibition but a marked downregulation of immunoregulatory interactions in the IZ of RPL patients. Through integrated single-cell chromatin accessibility and transcription factor occupancy analyses, we identified FOSL2 as a pivotal regulator orchestrating the spatial transformation of dNK1 cells. Decreased FOSL2 expression correlated with compromised IL-15-induced dNK1 cell transformation and diminished immunoregulatory capabilities. Our findings delineate the intricate spatial and regulatory architecture of immune tolerance within the human decidua, providing new insights into immune tolerance dysregulation in RPL.

## Graphical abstract

**Figure:**
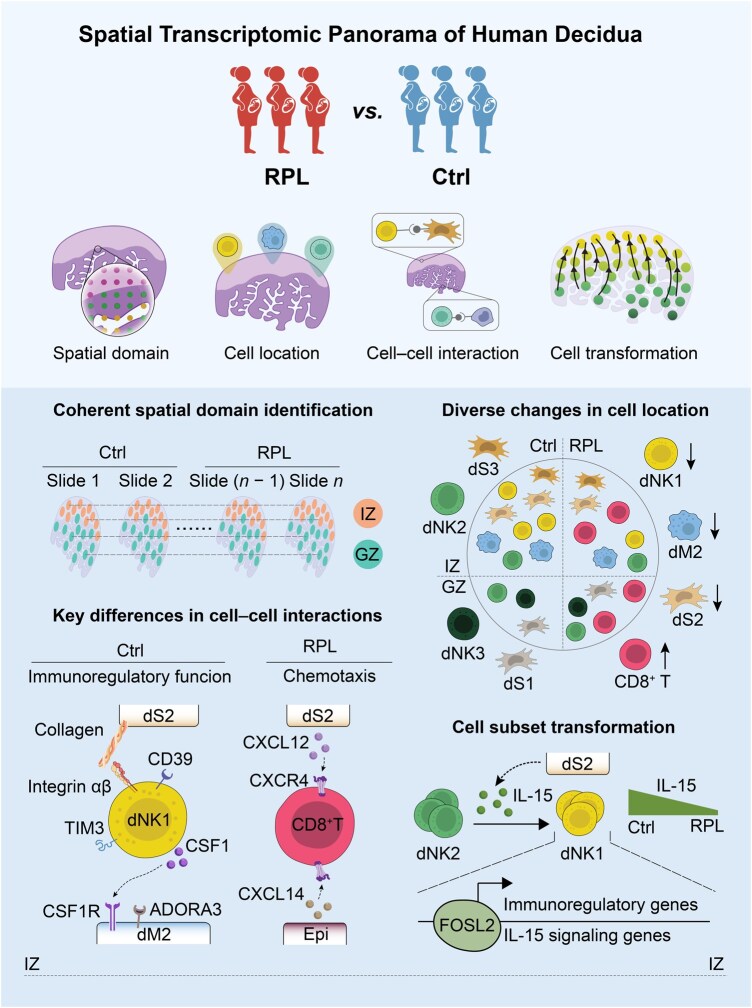

## Introduction

The human decidua, transformed from the endometrium during pregnancy, is a specialized tissue essential for providing nutritional support to the developing embryo [1]. The success of reproduction is intricately tied to the establishment of maternal immune tolerance to the semi-allogeneic embryo in the early stages of pregnancy. Recurrent pregnancy loss (RPL), defined as the loss of two or more consecutive pregnancies, is a common first-trimester complication affecting approximately 1%–5% of women worldwide [2]. While chromosomal abnormalities and uterine anatomical issues are known contributors, the underlying causes of nearly half of RPL cases remain elusive [3]. Independent studies have underscored the dysregulation of decidual immune homeostasis in cases of unexplained RPL [2,4−6], illuminating the potential link between disturbances in the decidual immune microenvironment and the adverse outcomes of pregnancy.

The human decidua exhibits a highly organized architecture in response to embryo implantation, comprising two primary functional layers: the compacta and the spongiosa [7]. The decidua compacta serves as the embryo’s implantation site and facilitates interactions with maternal decidual cells [8−10], and is enriched in decidualized stromal cells (dS2 and dS3). Conversely, the decidua spongiosa houses secretory glands vital for nourishing the embryo [11], and is enriched in undifferentiated dS1 cells. Recent advancements using single-cell RNA sequencing (scRNA-seq) have revealed the cellular diversity within the decidua and detailed immune cell composition changes in RPL patients [4,6,12]. Among the immune cells, decidual natural killer (dNK) cells are the most abundant and exhibit high heterogeneity. dNK1 cells are characterized by high expression of inhibitory receptors (KIR and TIM-3) and immunomodulatory molecules (CD39 and SPINK2) [12]. They function in guiding trophoblast differentiation [13] and enhancing angiogenesis [14], endowing dNK1 cells with immunoregulatory roles in uterine immune adaptation and fetal development. In contrast, dNK3 cells, marked by CD103 and CXCR4, display pronounced cytotoxicity against JEG3 and K562 cell lines and heightened responses in missing-self recognition assays [13,15]. In RPL patients, the frequency of immunoregulatory dNK1 cells is significantly reduced, while that of cytotoxic dNK3 cells is elevated. The proportions of T cells and decidual macrophages (dM) also show notable shifts. However, the lack of tissue information hinders the understanding of spatial organization and cell–cell interactions in the decidua of both healthy individuals and those with RPL.

During the first trimester, decidual immune cells, especially dNK cells, undergo notable phenotypic and functional changes to foster immune tolerance to the embryo [16]. Contemporary studies have sought to detail the transformation of other dNK cells to the dNK1 subset, in which the hindered transformation of dNK cells may drive altered immune cell compositions and imbalances in immune homeostasis witnessed in RPL patients [6,14]. This is supported by a recent study indicating that the transfer of dNK1 cells can rescue pregnancy failure and impaired placental development in a humanized mouse model [13]. However, these investigations fall short in discerning the extracellular signaling pathways and the intracellular transcriptional regulatory mechanisms that may modulate dNK cell phenotypes [17]. Consequently, the molecular intricacies steering dNK cell transformation, functionality, and spatial arrangement remain unclear.

Using 10x Visium spatial transcriptomics (ST) technology on human decidual samples from both healthy controls and RPL patients, we identified two spatial domains with consistent cell type compositions and gene expression patterns across ten samples, each aligned with a distinct decidual layer. By integrating scRNA-seq datasets, we pinpointed unique immunological niches between these domains. Specifically, the domain corresponding to the decidua compacta was enriched in immunoregulatory cell subsets and exhibited prominent cell–cell interactions. In contrast, the other domain was enriched in cytotoxic cell subsets. Notably, in RPL patients, the region-specific immunological niche associated with immune tolerance was disrupted, as reflected by the diminished distribution of immunoregulatory dNK cell subsets and their associated cell–cell interactions. Delving further into single-cell chromatin accessibility analysis via single-cell transposase-accessible chromatin sequencing (scATAC-seq), we identified FOSL2 as a pivotal regulator of dNK cell transformation within the decidua compacta. We subsequently demonstrated that knocking down *FOSL2* impaired IL-15-driven dNK cell transformation and its immunoregulatory function. Collectively, our research provides a spatially resolved landscape of the decidual immunological niche during early pregnancy and offers novel perspectives on the molecular dynamics associated with immune tolerance dysregulation in RPL patients.

## Results

### Identification of two coherent spatial domains in the human decidua

**Figure 1:**
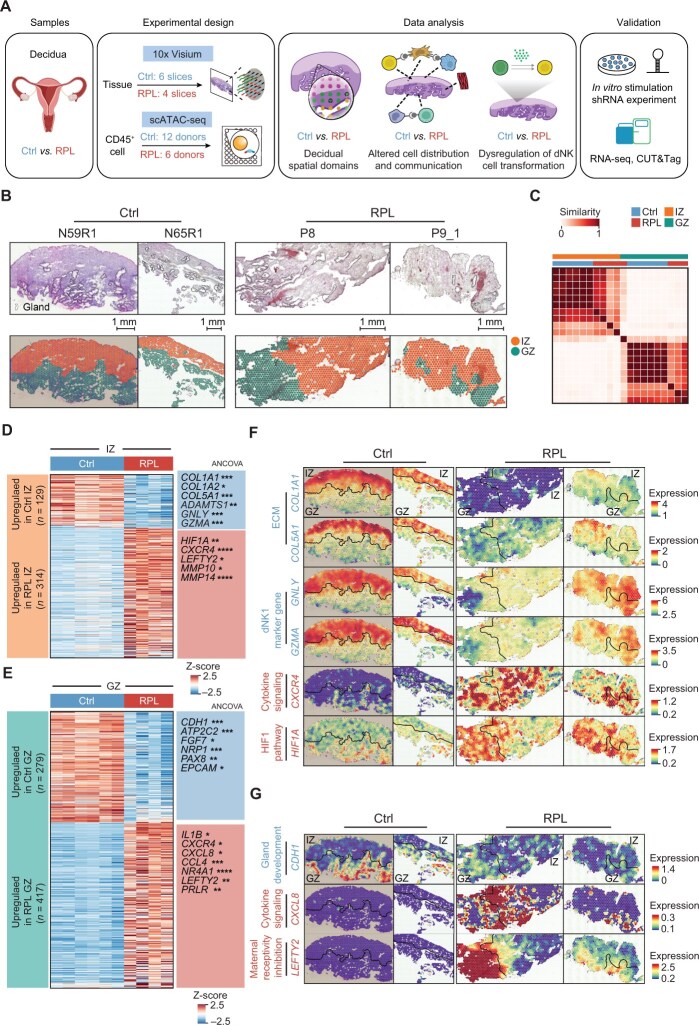
Spatially resolved transcriptomic atlas of human decidua in healthy donors and RPL patients **A**. Schematic overview of the study design. **B**. Bottom: Spatial domains (IZ and GZ) identified by SPACEL [20] in deciduas from healthy donors (N59R1 and N65R1) and RPL patients (P8 and P9_1). Top: Corresponding histological images with dotted lines outlining the glandular regions. Scale bar, 1 mm. **C**. Jaccard similarity coefficient of signature genes for spatial domains among the ten ST sections. **D**. and **E**. Heatmap showing the DEGs in IZ (D) or GZ (E) between healthy donors and RPL patients. Statistical significance of genes annotated alongside the heatmap was assessed using two-sided ANCOVA (*, *P* < 0.05; **, *P* < 0.01; ***, *P* < 0.001; ****, *P* < 0.0001). **F**. Spatial expression of dNK1 marker genes and DEGs related to ECM, cytokine signaling, and HIF1 signaling in the decidua of healthy donors and RPL patients. **G**. Spatial expression of DEGs related to gland development, cytokine signaling, and maternal receptivity inhibition in the decidua of healthy donors and RPL patients. Ctrl, control; RPL, recurrent pregnancy loss; scATAC-seq, single-cell transposase-accessible chromatin sequencing; shRNA, short hairpin RNA; RNA-seq, RNA sequencing; CUT&Tag, cleavage under targets and tagging; IZ, implantation zone; GZ, glandular-secretory zone; ST, spatial transcriptomics; ANCOVA, analysis of covariance; ECM, extracellular matrix; dNK, decidual natural killer; DEG, differentially expressed gene.

We employed the 10x Visium ST sequencing method to analyze ten decidual tissue sections obtained from first-trimester pregnancies. Of these, six sections were derived from six healthy controls, and the remaining four were from three RPL patients (**Figure 1**A). The demographic data indicated a donor age range of 19–39 years for both healthy controls and RPL patients. The mean gestational ages stood at 6.77 weeks for controls and slightly higher at 7.89 weeks for RPL patients (Table S1). Hematoxylin and eosin (H&E) staining indicated that each tissue section consisted of two distinct anatomical decidual layers: the decidua compacta, located proximal to the luminal epithelium, and the subjacent decidua spongiosa, characterized by hypersecretory glands (Figure 1B, Figure S1A). We initially employed StarDist [18] to process the histological images of the 10x Visium data and calculated the number of cells per spot. After eliminating ST spots with a cell number ≤ 0, our dataset consisted of 14,282 individual spots. On average, 4906 genes were expressed per spot (Table S2).

To delineate the functional domains in the decidua, we employed Cell2location [19] to deconvolute the cell type composition of each spot in the ST data using the previously obtained scRNA-seq profiles as a reference [4,6,12]. We then utilized the Splane module of SPACEL [20] and identified two spatial domains, named implantation zone (IZ) and glandular-secretory zone (GZ), which were coherent across all ten decidual sections studied (Figure 1B and C, Figure S1A). Notably, these domains exhibited morphologically layered distribution characteristics: the IZ was distributed in the upper layer near the luminal surface, where embryo implantation occurs, and the GZ was distributed in the lower layer near the basal layer, containing secretory glands. We then visualized the expression of stromal cell subset marker genes [12] in each slice and observed that the IZ had high expression of genes such as *PRL*, *CYP11A1*, and *IGFBP2*, which are typically associated with stromal cell subsets in the decidua compacta (dS2 and/or dS3 cells). On the other hand, the GZ showed high expression of stromal cell marker genes representing the decidua spongiosa (dS1), such as *ACTA2* and *TAGLN* (Figure S1B and C). Collectively, these findings suggest that the IZ and GZ are indicative of the decidua compacta and spongiosa, respectively.

To further investigate the biological significance of the two spatial domains in both healthy and RPL states, we performed pairwise analyses between these domains under the two states. This revealed 804 differentially expressed genes (DEGs) in the ST spots, with |log_2_ fold change (FC)| ≥ 0.5 and *P* < 1 × 10^−10^ as the cutoff criteria (Figure 1D and E; Table S3). Gene Ontology (GO) analysis of these DEGs indicated that genes upregulated in the normal IZ were significantly enriched in terms associated with extracellular matrix (ECM) organization, such as “Type I collagen synthesis” (Figure 1D and E, Figure S1D). Marker genes of dNK1 cells with growth-promoting [21] and immunoregulatory capabilities [12] were also found to be upregulated in the normal IZ. This finding suggested a role in facilitating embryo implantation within the compacta layer of the decidua. Yet, in RPL patients, we observed a remarkable expression reduction of multiple ECM genes like *COL1A1* and *COL5A1* as well as dNK1 marker genes *GNLY* and *GZMA*, which was confirmed by analysis of covariance (ANCOVA) with gestational age included as a covariate (Figure 1D and F). Conversely, there was a significant upsurge in genes associated with the HIF1 pathway (notably *HIF1A*) and cell chemotaxis (such as *CXCL8*) in the RPL sections (Figure 1G). Meanwhile, genes upregulated in the normal GZ were chiefly linked to gland development, echoing glandular progression in the spongiosa layer (Figure S1D). However, genes known to obstruct maternal receptivity [22], such as *LEFTY2*, showed a notable increase in the GZ of RPL sections (Figure 1G). These results suggest that the alterations of the decidual immune states in the IZ and GZ may contribute to different aspects of RPL pathogenesis.

### Disrupted spatial distribution of immunoregulatory and cytotoxic cells in RPL patients

**Figure 2:**
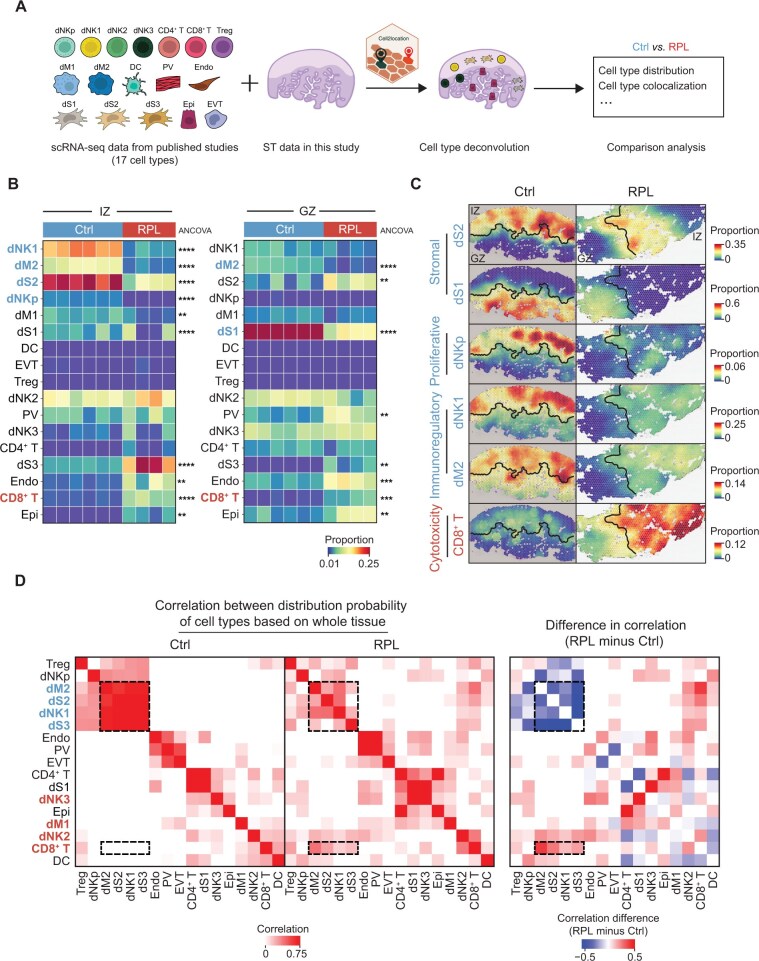
Distinct cellular distribution and colocalization in spatial domains of healthy donors and RPL patients **A**. Deconvolution strategy for determining cell type distribution probability by integrating ST data in this study and public scRNA-seq datasets using Cell2location [19]. **B**. Normalized distribution probabilities (proportions) of the 17 cell types in the IZ (left) and GZ (right) for healthy donors and RPL patients. The original distribution probability given by Cell2location for each cell type was normalized by the proportion of the cell type in the single-cell data, resulting in the normalized distribution probability. Statistical significance was determined by two-sided ANCOVA (**, *P* < 0.01; ***, *P* < 0.001; ****, *P* < 0.0001). **C**. Spatial distribution probabilities of dS1, dS2, dNKp, dNK1, dM2, and CD8^+^ T cells in the decidua of healthy donors and RPL patients. **D**. Within-spot Pearson correlation analysis of cell type distribution probabilities showing distinct patterns of cellular colocalization in healthy donors (left) and RPL patients (middle). The heatmap on the right shows the difference in pairwise cell type Pearson correlation coefficients between RPL patients and healthy donors. The dashed boxes represent the significantly altered correlations of cell type pairs in RPL patients compared to healthy donors. scRNA-seq, single-cell RNA sequencing; dS, decidual stromal; dM, decidual macrophage; dNKp, proliferating dNK cell; Treg, regulatory T cell; DC, dendritic cell; PV, perivascular cell; Endo, endothelial cell; Epi, epithelial glandular cell; EVT, extravillous trophoblast.

Given that tissue function is closely linked to the spatial distribution of constituent cell subsets [23], our further investigation focused on understanding the two-dimensional (2D) spatial distribution of cell subsets and their alterations in RPL patients. To address this, we utilized publicly available scRNA-seq datasets that characterized 17 major cell types, including dS1–dS3, dNK1–dNK3, and dM1–dM2 cells, in first-trimester decidual tissue from healthy and RPL individuals [4,6,12]. We performed spatial cell type deconvolution with Cell2location [19], yielding a spatial distribution for each cell type in 2D space (**Figure 2**A). Three stromal subsets were distributed in their anticipated anatomical locations, with dS2 and dS3 cells predominantly located in the IZ and dS1 cells preferentially positioned in the GZ (Figure S1C), consistent with a prior study employing immunohistochemistry and single-molecule fluorescence *in situ* hybridization [12], supporting the reliability of our cell type deconvolution results.

In the normal decidua, we observed a prominent distribution of two immune subsets, dNK1 and dM2 cells in the IZ (Figure 2B), which are known to promote maternal immune tolerance to the embryo [12]. Consistent with our findings, a prior immunofluorescence study demonstrated that CD39^+^CD56^+^ dNK1 cells were predominantly localized to the compacta layer [5], confirming their spatial positioning at the maternal–fetal interface. Remarkably, we found a significant reduction of dNK1 and dM2 cells in the IZ of RPL patients (Figure 2B and C, Figure S2A). The dNK3 cells, which exhibit cytotoxic and immune-activated properties [15], were exclusively found in the GZ (Figure 2B). Moreover, the cytotoxic CD8^+^ T cells, which were sparsely distributed in the normal decidua, exhibited a broad increase in both the IZ and GZ of RPL patients (Figure 2C). For decidual stromal (dS) cells, we observed that decidualized dS2 cells and undifferentiated dS1 cells were remarkably decreased in the IZ and GZ of RPL patients, respectively. To further validate these findings, we applied an alternative cell type deconvolution algorithm, Tangram [24], and obtained similar results (Figure S2B and C).

To investigate the colocalization of different cell types in 2D space, we calculated the Pearson correlation between paired cell types at each spatial spot. Cell types distributed within the same spatial domain were found to be significantly colocalized (Figure 2D, Figure S2D). For instance, in the normal decidua, there was notable colocalization among dM2, dNK1, dS2, and dS3 cells in the IZ. However, in RPL patients, we observed markedly reduced colocalization among these cells in the IZ (Figure 2C and D). On the other hand, the cytotoxic CD8^+^ T cell displayed elevated colocalization with multiple cell types in both the IZ and GZ, particularly in the IZ of RPL patients (Figure 2D). Together, these findings indicate an aberrant stromal cell distribution and a disrupted immunological niche in RPL, specifically a decrease of immunoregulatory cells in the IZ and an increase in cytotoxic cells in both domains.

### Cell–cell interactions maintaining maternal immune tolerance in the decidua compacta are decreased in RPL patients

**Figure 3:**
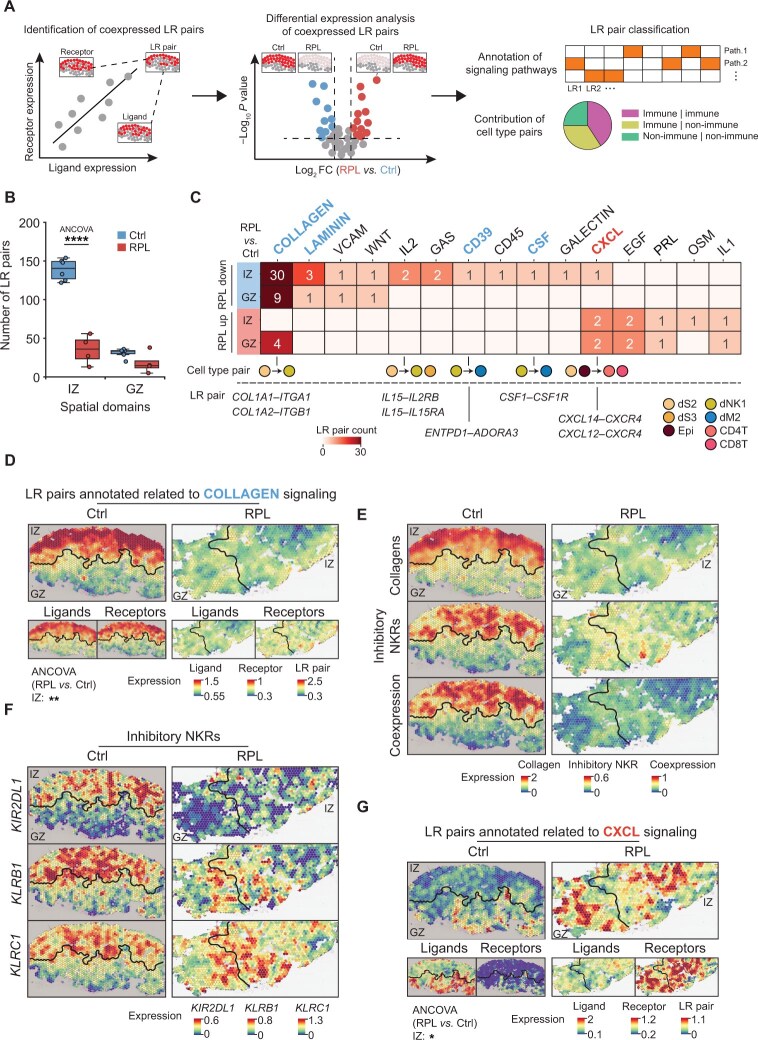
Comparative analysis of cell–cell interactions in spatial domains of healthy donors and RPL patients **A**. Strategy for analyzing cell–cell interactions based on ST data. **B**. Comparison of the number of coexpressed LR pairs in spatial domains between healthy donors and RPL patients. Statistical significance was determined by two-sided ANCOVA (****, *P* < 0.0001). **C**. Classification of differentially expressed LR pairs into functionally related signaling pathways. The number of LR pairs enriched in a given pathway and the cell type pair with the highest expression of the corresponding LR pair are indicated. Only spatially colocalized cell types were considered here (see Materials and methods). **D**. Spatial average expression of LR pairs associated with collagen signaling in the decidua of healthy donors and RPL patients. Statistical significance of the LR average expression was determined by two-sided ANCOVA (**, *P* < 0.01). **E**. Spatial average expression of genes encoding collagens (top) and inhibitory NKRs (middle) in the decidua of healthy donors and RPL patients. Spatial coexpression of genes encoding collagens and inhibitory NKRs is shown at the bottom. **F**. Spatial average expression of genes encoding inhibitory NKRs in the decidua of healthy donors and RPL patients. **G**. Spatial average expression of LR pairs associated with CXCL signaling in the decidua from healthy donors and RPL patients. Statistical significance of the LR average expression was determined by two-sided ANCOVA (*, *P* < 0.05). LR, ligand–receptor; FC, fold change; Path., pathway; NKR, natural killer cell receptor.

Next, we explored the critical ligand–receptor (LR) pairs and signaling pathways that contribute to the disruption of local cellular interactions within decidual spatial domains in both healthy controls and RPL patients. First, we calculated the spatial coexpression correlation coefficient (ρ) for each LR pair in the CellPhoneDB database [12] to search for spatially coexpressed LR pairs (**Figure 3**A). Our analysis revealed 170 coexpressed LR pairs in the normal decidua (ρ > 0, *P* < 0.05), 139 of which were distributed in the IZ and 31 in the GZ (Figure 3B, Figure S3A), suggesting that the IZ is a cell–cell interaction hotspot in decidua. In contrast, only 53 coexpressed LR pairs were identified in the decidua of RPL patients, 35 of which were distributed in the IZ and 18 in the GZ, suggesting that the reduction in coexpressed LR pairs in RPL patients is mainly manifested in the IZ (*P* < 0.05).

We then conducted differential expression analysis of all the coexpressed LR pairs between healthy controls and RPL patients (|log_2_ FC| ≥ 0.1, *P* < 1 × 10^−10^) (Figure 3C, Figure S3B and C). We found that LR pairs associated with collagen signaling were predominantly downregulated in the IZ of RPL patients (mean |log_2_ FC| = 0.9, adjusted *P* value < 1 × 10^−5^) (Figure 3C and D, Figure S3D and E). LR pairs in the collagen signaling (*e.g.*, *COL1A1‒ITGA1* and *COL1A2‒ITGB1*) are known to promote the retention and prevent excessive immune responses of dNK cells by inducing the expression of inhibitory NK cell receptors (NKRs) [25]. The expression of multiple inhibitory NKRs (*e.g.*, *KIR2DL1*,* KLRB1*, and* KLRC1*) was significantly decreased in the IZ of RPL patients (Figure 3E and F, Figure S4A), suggesting that dNK1 cell-mediated maternal immune tolerance is impaired in the disease state. Furthermore, the CSF and CD39 pathways, primarily mediated by dNK1 cells to promote chemotaxis and suppress the proinflammatory response of macrophages [26], were also downregulated in the IZ of RPL patients (Figure S4B and C).

In contrast, CXCL pathway-associated LR pairs were upregulated in both the IZ and GZ of RPL patients (Figure 3G). CXCL ligands (*CXCL12* and* CXCL14*) exhibited high expression in glandular epithelial cells and stromal cells in the decidua compacta, and their receptor *CXCR4* was significantly upregulated in T cells of RPL patients (Figure S4D). Since we previously observed increased colocalization of dS2 cells with CD8^+^ T cells (Figure 2C and D), we next analyzed the spatial distribution of these cells and the spatial expression of CXCL pathway genes. We found that CD8^+^ T cells were distributed around the glands and dS2 cells with a strong CXCL signal in RPL patients (Figure S4E), suggesting enhanced chemotaxis of CD8^+^ T cells in the IZ, consequently exacerbating the disturbance of maternal immune tolerance.

### IL-15 promotes dNK1 cell accumulation and immunoregulatory gene expression in the decidua compacta

**Figure 4:**
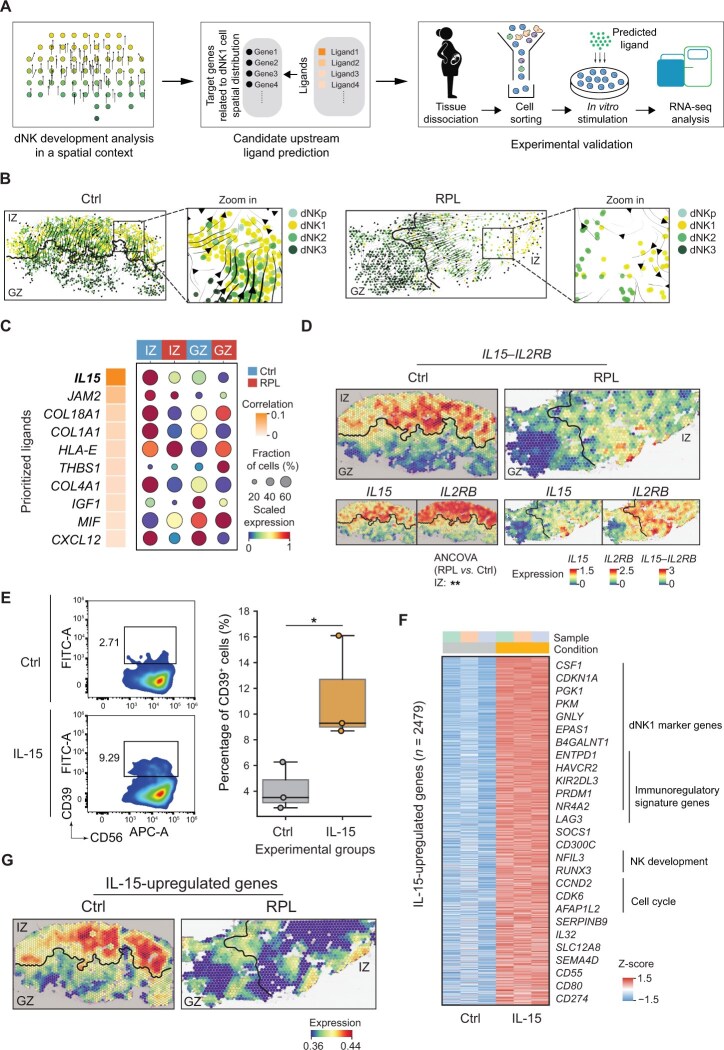
dNK cell spatial transformation in the compacta layer **A**. Strategy for dNK cell spatial transformation and upstream ligand analysis. **B**. RNA velocity stream of dNK cells at spatial resolution. Tangram [24] was utilized to map individual cells by integrating scRNA-seq and ST data, followed by RNA velocity analysis. **C**. Outcome of NicheNet’s ligand activity prediction [30] on genes positively correlated with the distribution probability of dNK1 cells (*n* = 204). The dotplot in the right panel indicates the expression of each ligand in the spatial domains of healthy donors and RPL patients. **D**. Spatial expression of *IL15*, *IL2RB*, and *IL15‒IL2RB* in the decidua of healthy donors and RPL patients. Statistical significance of the LR average expression was determined by two-sided ANCOVA (**, *P* < 0.01). **E**. Percentage of CD39^+^ dNK1 cells after stimulation of CD18^+^ dNK2 cells with IL-15 *in vitro* for 2 days compared to that in the control group (*n* = 3 for each group). Statistical significance was determined by a two-sided Wilcoxon rank-sum test (*, *P* < 0.05). **F**. Heatmap showing upregulated genes (*n* = 2479) following IL-15 stimulation assessed by RNA-seq analysis. **G**. Spatial average expression of IL-15-upregulated genes in the decidua of healthy donors and RPL patients.

Considering the pivotal role of dNK1 cells in maintaining immune tolerance at the maternal–fetal interface, we next investigated the spatiotemporal transformation of dNK cell subsets to reveal the molecular mechanisms underlying the reduction in dNK1 cells in RPL patients. To achieve this, we employed Tangram [24] to resolve the spatial location of individual dNK cells from scRNA-seq data, followed by RNA velocity analysis [27] to uncover the spatial transforming trajectory of dNK cells (**Figure 4**A). In normal decidua, we observed a clear trend for dNK2 cells to transform toward dNK1 cells in the IZ; however, this transformation potential was significantly reduced in RPL patients (*P* < 0.05; Figure 4B, Figure S5A–C). To further support this finding, we utilized veloVI [28] to estimate RNA velocity uncertainty, and then integrated it with partition-based graph abstraction [29] for trajectory confidence assessment. This analysis confirmed a robust transition from dNK2 to dNK1 cells in the IZ of healthy decidua (Figure S5D). Furthermore, a permutation test based on cell label shuffling demonstrated that this transformation trajectory was significant in healthy donors but not in RPL patients (*P* < 0.01; Figure S5E and F).

To explore the extracellular signals potentially driving local transformation to dNK1 cells in the IZ, we surveyed genes that exhibited a positive association with dNK1 cell distribution. By intersecting these genes with dNK cell signature genes, we obtained 204 genes that may be relevant to dNK1 cell spatial transformation. We selected these genes as targets for NicheNet [30] to predict upstream ligands that regulate their expression (Figure 4A). Our analysis identified 10 upstream ligands, including *IL15*,* COL18A1*, and *COL1A1* (Figure 4C). Notably, *IL15* emerged as the top-ranked ligand, specifically expressed in the normal IZ. The receptor of *IL15*, *IL2RB*, was also highly expressed in dNK1 cells according to scRNA-seq data (Figure S6A). We examined the relationship between the spatial distribution of dNK cell subsets and IL-15 signaling by analyzing Pearson correlation coefficients between the spatial distribution probability of each dNK cell subset and the spatial gene expression of *IL15* and *IL2RB*. In the decidua of healthy controls, we observed that the dNK1 subset showed a strong correlation with the expression of *IL15* and *IL2RB* (Figure S6B and C). However, this correlation was significantly attenuated in RPL patients (Figure S6B and C). Similar to the reduction of dNK1 cell distribution in the IZ of RPL patients, the expression of the *IL15* and *IL2RB* genes was also significantly decreased in the IZ of RPL patients (Figure 4D). These results suggest that IL-15 signaling may be involved in regulating the transformation of dNK1 cells from dNK2 cells within the IZ.

To further investigate the impact of IL-15 signaling on promoting the transformation of dNK2 cells into dNK1 cells, we conducted an experiment in which CD39^−^CD18^+^ dNK2 cells were sorted and subjected to IL-15 stimulation *in vitro* (Figure 4A). We observed successful transformation of these cells into CD39^+^ dNK1 cells upon IL-15 stimulation (Figure 4E). Moreover, we performed RNA-seq analysis on IL-15-stimulated cells and identified significant upregulation of multiple dNK1 marker genes (*e.g.*, *CSF1*, *PKM*, *CDKN1A*, and *EPAS1*) and various immunoregulatory signature genes (*LGALS1*, *KIR2DL3*, *HAVCR2*, and *ENTPD1*) (Figure 4F, Figure S6D; Table S4). These genes were predominantly expressed in the IZ of normal decidua and exhibited a remarkable decrease in expression in RPL patients (Figure 4G). Thus, these findings underscore the critical role of IL-15 signaling in promoting the accumulation of dNK1 cells in the compacta layer and therefore enhancing the immunoregulatory capabilities of dNK cells.

### FOSL2 is a transcriptional regulator of dNK1 cell transformation

**Figure 5:**
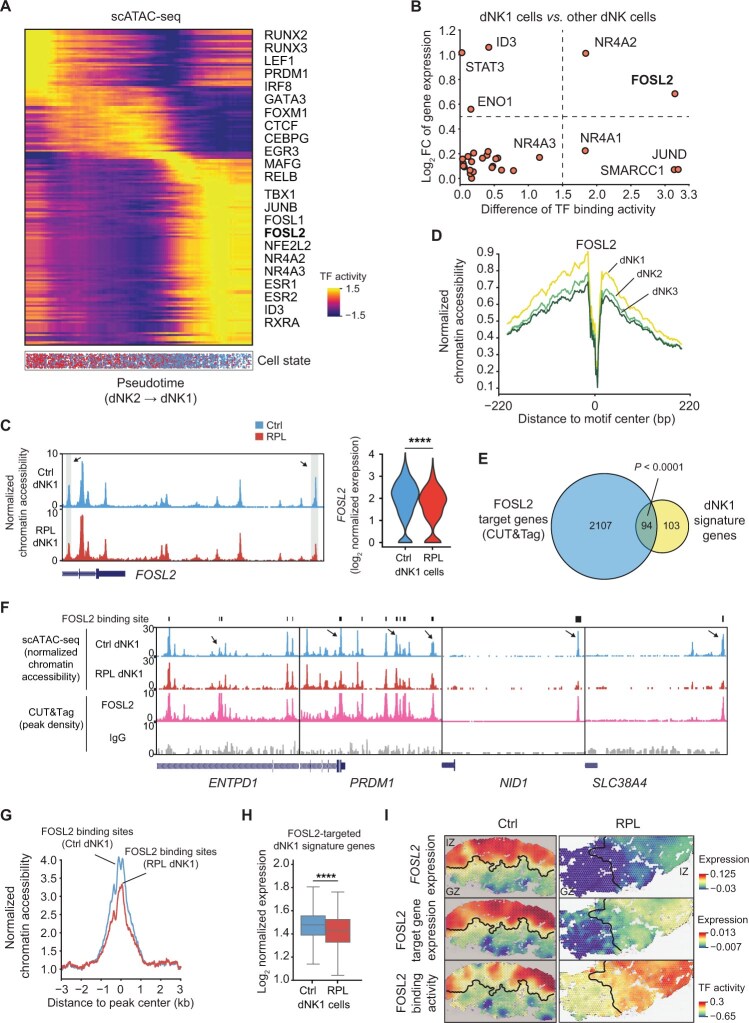
Multi-omics analysis revealed FOSL2 as a candidate regulator of dNK cell spatial transformation **A**. Changes in the binding activity of TFs along the pseudotime trajectory of dNK cells (from dNK2 cells to dNK1 cells). **B**. Scatter plot illustrating the difference in TF binding activity and log_2_ FC in TF expression between dNK1 cells and other dNK cells. **C**. Chromatin accessibility and gene expression of FOSL2 in dNK1 cells from healthy controls and RPL patients. The arrow and gray shading in the genome browser tracks highlight the open regions that were more accessible in dNK1 cells from healthy donors than in those from RPL patients. Statistical significance was determined by a two-sided Student’s *t*-test (****, *P* < 0.0001). **D**. TF footprinting analysis showing average chromatin accessibility around the FOSL2 motif center in dNK cell subsets. **E**. Venn diagram showing the overlap between the dNK1 signature genes and FOSL2 target genes detected by CUT&Tag. Statistical significance was determined by the chi-squared test. **F**. Normalized UCSC genome browser tracks of chromatin accessibility from scATAC-seq and FOSL2 occupancy from CUT&Tag profiling at the *ENTPD1*, *PRDM1*, *NID1*, and *SLC38A4* loci. The arrows highlight the FOSL2-occupied open regions that were more accessible in dNK1 cells from healthy donors than in those from RPL patients. **G**. Average chromatin accessibility around the center of FOSL2 binding sites in dNK1 cells from healthy donors and RPL patients. **H**. Box plot showing the average expression of FOSL2-targeted dNK1 signature genes in dNK1 cells from healthy donors and RPL patients. Statistical significance was determined by a two-sided Student’s *t*-test (****, *P* < 0.0001). **I**. Spatial patterns of *FOSL2* expression, FOSL2 target gene expression, and FOSL2 binding activity in the decidua of healthy donors and RPL patients. The TF expression (binding activity) in each spot was calculated by summing the product of the distribution probability of the three dNK cell subsets in the spot and the corresponding expression (binding activity) in the single-cell data. The average expression of FOSL2 target genes detected by CUT&Tag in the scRNA-seq data was used as the FOSL2 target gene expression of each dNK cell subset, and the FOSL2 target gene expression of each spot was subsequently calculated using the same strategy as above. TF, transcription factor; UCSC, University of California, Santa Cruz.

To elucidate the transcriptional regulatory mechanism underlying dNK1 cell transformation, we conducted scATAC-seq analysis of purified CD45^+^ immune cells obtained from first-trimester decidual samples of 12 healthy controls and 6 RPL patients (Figure 1A; Table S1). Our analysis identified 11 distinct immune cell subsets, including three subsets of dNK cells (dNK1, dNK2, and dNK3), two subsets of macrophages, three subsets of T cells, dendritic cells, natural killer T (NKT) cells, and group 3 innate lymphoid cells (ILC3s) (Figure S7). Using Palantir [31], we inferred the trajectory of dNK cells, which confirmed the observed transformation trend starting from dNK2 cells and progressing toward dNK1 cells (Figure S8A). Subsequently, we employed chromVAR [32] to predict the binding activity of transcription factors (TFs) in each cell and successfully identified TFs known to play crucial roles in dNK cell development and survival (**Figure 5**A), including RUNX3 [33] and RUNX2 [34], which exhibited enrichment at the early stage of the trajectory.

To further explore the TFs that potentially regulate dNK1 cell transformation, we assessed the expression and binding activity of each TF in dNK1 cells and compared them to those in other dNK subsets using a combination with scRNA-seq data. We found that FOSL2, a member of the AP-1 family known for its regulatory role in NK cell maturation [35], exhibited a progressive increase in binding activity along the trajectory, with the highest binding activity and expression observed in dNK1 cells compared to other dNK cells (Figure 5A and B). Furthermore, we observed a significant reduction in the accessibility and gene expression of FOSL2 in dNK1 cells from RPL patients (Figure 5C). Intriguingly, we also discovered enhanced DNA accessibility and a stronger TF occupancy footprint surrounding FOSL2 motifs in dNK1 cells than in other subsets of dNK cells (Figure 5D).

We then investigated the genomic occupancy of FOSL2 by conducting cleavage under targets and tagging (CUT&Tag) experiments specifically on dNK1 cells. Our analysis revealed that approximately half of the dNK1 cell signature genes, including *ENTPD1*, *PRDM1*, *NID1*, and *SLC38A4*, were bound by FOSL2 (Figure 5E and F). Furthermore, the chromatin accessibility and expression of these genes were markedly diminished in RPL patients (Figure 5F‒H). By integrating scATAC-seq and scRNA-seq data with ST data, we observed that FOSL2 exhibited elevated binding activity in the normal IZ. Meanwhile, *FOSL2* and its target genes were found to be specifically and highly expressed in the normal IZ; however, their expression was significantly reduced in RPL patients (Figure 5I). These results suggest that FOSL2 plays a pivotal role in driving the maturation and immunoregulatory capabilities of dNK1 cells within the normal IZ, whose function was impaired in RPL patients.

### *FOSL2* knockdown impairs IL-15-driven dNK1 cell transformation

**Figure 6:**
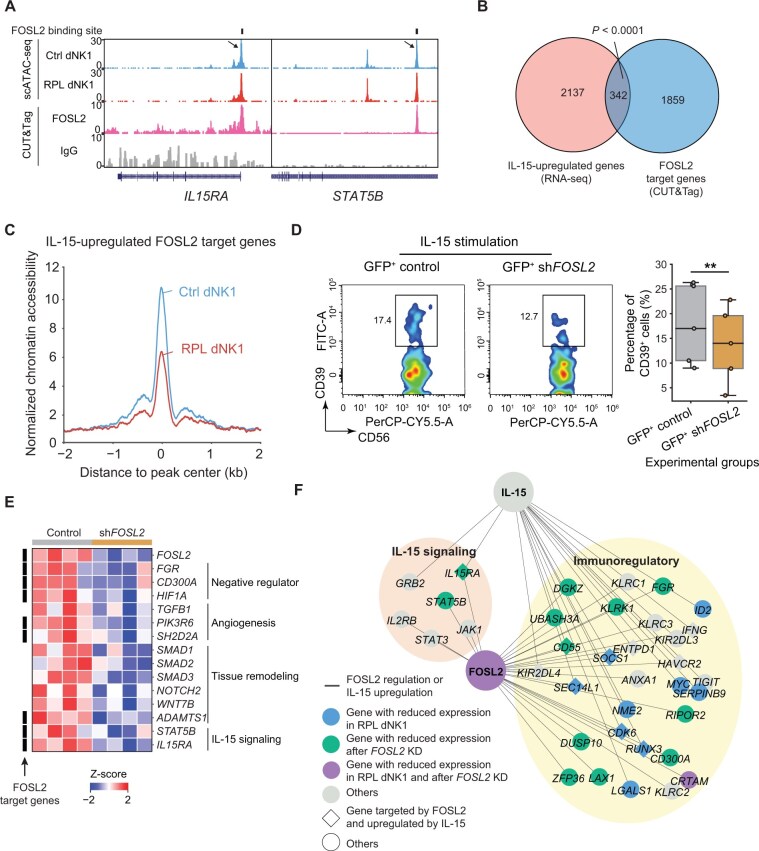
Loss of FOSL2 activity impairs IL-15-mediated dNK cell transformation **A**. Normalized UCSC genome browser tracks of chromatin accessibility from scATAC-seq and FOSL2 occupancy from CUT&Tag profiling at the *IL15RA* and *STAT5B* loci. The arrows highlight the FOSL2-occupied open regions that were more accessible in dNK1 cells from healthy donors than in those from RPL patients. **B**. Venn diagram showing the overlap between the IL-15-upregulated genes detected by RNA-seq and FOSL2 target genes detected by CUT&Tag. Statistical significance was determined by the chi-squared test. **C**. Average chromatin accessibility around the center of FOSL2-bound peaks related to IL-15-upregulated genes in dNK1 cells from healthy donors and RPL patients. **D**. Percentage of CD39^+^ dNK1 cells among GFP^+^ control and sh*FOSL2* lentivirus-infected cells after 2 days of IL-15 stimulation *in vitro* (*n* = 5 for each group). Statistical significance was determined by a paired Student’s *t*-test (**, *P* < 0.01). **E**. Heatmap showing the Z-scaled gene expression of specific DEGs in control lentivirus-infected cells (*n* = 4) and sh*FOSL2* lentivirus-infected cells (*n* = 4). **F**. Signaling network integrating IL-15 signaling molecules and FOSL2 target genes, focusing on the regulation of immunoregulatory functions. The edges represent FOSL2 regulation or IL-15 upregulation. The nodes represent genes targeted by FOSL2 and/or downstream of IL-15 signaling. Downstream genes of IL-15 signaling were defined as those upregulated by IL-15 stimulation and/or belonging to the “REACTOME_INTERLEUKIN_15_SIGNALING” term in the MSigDB database (https://www.gsea-msigdb.org/gsea/msigdb). The color of each node indicates the differential expression type of the gene. The shape of each node indicates the type of gene regulation. KD, knockdown.

Among the FOSL2 target genes, we observed IL-15 receptor genes and multiple downstream genes of the IL-15 signaling pathway, such as *IL15RA* and *STAT5B* (**Figure 6**A, Figure S8B). Specifically, 16% of the IL-15-upregulated genes were found to be targeted by FOSL2 (*P* < 0.0001; Figure 6B). The chromatin accessibility of these genes in dNK1 cells was also decreased in RPL patients (Figure 6C). These results suggest that FOSL2 may work together with IL-15 to drive the transformation of dNK1 cells.

To test this hypothesis, we knocked down *FOSL2* by transfecting CD39^−^CD18^+^ dNK2 cells with short hairpin RNA (shRNA) lentivirus, followed by IL-15 stimulation for 2 days (Figure S9A and B). Real-time quantitative polymerase chain reaction (RT-qPCR) analysis of lentivirus-infected dNK cells revealed substantially decreased expression of *FOSL2* in the *FOSL2*-targeting group compared with the control group, providing compelling evidence of successful *FOSL2* knockdown (KD) (Figure S9C). We then found that *FOSL2* KD resulted in a significant decrease in the proportion of CD39^+^ dNK1 cells compared with the control group (Figure 6D), indicating that the loss of FOSL2 impairs IL-15-mediated dNK1 cell transformation.

To explore the interplay between FOSL2 and IL-15, we conducted RNA-seq analysis on IL-15-stimulated control and *FOSL2* KD dNK2 cells. Differential expression analysis between the control and KD groups revealed four clusters of genes, related to IL-15 signaling (*STAT5B* and *IL15RA*), angiogenesis (*TGFB1*) [36], tissue remodeling (*NOTCH2* and *SMAD1*) [37,38], and NK cell cytotoxicity inhibition (*HIF1A*, *FGR*, and *CD300A*) [39−41] (Figure 6E, Figure S9D and E). The overall expression of IL-15-upregulated genes was also decreased after *FOSL2* KD (Figure S9F and G), demonstrating that FOSL2 is essential for IL-15 signaling that drives the transition to dNK1 identity.

To further support the regulatory role of FOSL2 in dNK1 cell transformation and function, we utilized SCENIC+ [42] to simulate *FOSL2* overexpression in dNK cells from RPL patients, which significantly upregulated dNK1 signature genes (*e.g.*, *CSF1*, *CDKN1A*, and *STAT3*) and immunoregulatory marker genes (*e.g.*, *HAVCR2*, *KIR2DL1*, and *KIR2DL3*) (Figure S10A and B). Notably, trajectory analysis revealed a shift from dNK2 cells to dNK1-like states upon *FOSL2* overexpression (Figure S10C). Moreover, dNK2 cells with high *FOSL2* expression in RPL patients exhibited elevated expression of dNK1 signature genes (*e.g.*, *CSF1* and *TNFRSF18*) compared to counterparts with low *FOSL2* expression (Figure S10D–F). Taken together, these results underscore the crucial regulatory role of FOSL2 in the IL-15-mediated transformation and immune tolerance of dNK cells (Figure 6F).

## Discussion

The spatial organization and interactions of cells within multicellular tissues, such as the decidua, play crucial roles in normal physiological processes. In this study, we systematically surveyed the spatial distribution of human decidual cells in both healthy donors and RPL patients during early pregnancy. Our analysis revealed the presence of two distinct spatial domains characterized by coherent expression patterns and histology across diverse samples. Notably, these domains exhibited significant alterations in the distribution and interactions of immune cell subsets in RPL patients, particularly evidenced by a reduction in the dNK1 cell subset known for its immunoregulatory capabilities. The observed accumulation of dNK1 cells was found to be regulated by FOSL2, and FOSL2 dysregulation was linked to impaired immune tolerance in RPL patients. The experimental evidence provided in this study supports the regulatory role of FOSL2 in IL-15-mediated dNK cell transformation, further underscoring its significance in the context of RPL.

Recent investigations employing spatial omics at the maternal−fetal interface have shed light on the spatial correlation between the distribution of specific immune cells and embryonic invasion [43], as well as vascular remodeling [44] during the first trimester. The observed changes in the spatial distribution and interactions of immune cells in RPL patients underscore the substantial role of immunological aberrations in disease status [4–6]. For instance, dNK1 cells express CD39, which can mediate the production of immunosuppressive adenosine. The decreased dNK1 cell population, coupled with the increased presence of CD8^+^ T cells in the decidua compacta of RPL patients, may fail to effectively restrain the activity of CD8^+^ T cells, thereby exacerbating embryo rejection in RPL. Consequently, the methodology employed in this study holds the potential for investigating the underlying pathogenic mechanisms of various pregnancy complications.

IL-15 is known to undergo upregulation during the implantation window and has been reported to facilitate the proliferation and differentiation of dNK cells [14]. Our analysis revealed that IL-15 is predominantly expressed in the dS cell subset in the compacta layer. The observed reduction in IL-15 expression within the compacta layer of RPL patients is associated with impaired dNK cell transformation and compromised immunomodulatory function. Notably, *IL15* expression exhibits no significant changes in the dS2 cells in the disease state, suggesting that the reduction in *IL15* expression was primarily due to the diminished distribution of these stromal cells. This finding was corroborated by a single-cell study indicating a reduced proportion of dS2 cells in RPL patients [4]. Moreover, a recent study conducted in abortion-prone mice elucidated that dysfunctional and spatially disordered stromal cells disrupted decidual hub specification, ultimately leading to miscarriage [45].

Understanding transcriptional regulatory programs is crucial for comprehending the mechanisms underlying biological processes, such as cell development and tissue generation. We integrated scATAC-seq data and identified key TFs involved in regulating the transformation and immunomodulatory capabilities of dNK cells. The transformation of dNK2 cells to dNK1 cells was accompanied by enhanced binding activity of FOSL2, a downstream target of IL-15 signaling mediated by the TF STAT5 [46], suggesting a potential IL-15 signaling–FOSL2 axis that regulates dNK transformation. A typical dNK1 marker gene, *CSF1*, is both targeted by FOSL2 and upregulated by IL-15. *CSF1* encodes macrophage colony-stimulating factor (M-CSF), which is secreted specifically by dNK1 cells to regulate the early differentiation of trophoblast stem cells. Supplementation with human recombinant M-CSF or dNK1 cells efficiently reverses the pregnancy failure in mice [13], further emphasizing the important role of IL-15–FOSL2 signaling axis in supporting successful pregnancy. While our KD experiment and computational analysis provide supportive evidence for the role of FOSL2 in dNK1 cell transformation and function, future experimental validation in primary dNK cells will be needed to support the necessity of FOSL2 in driving dNK1 cell transformation. Aside from FOSL2, NR4A2 exhibited significant regulatory activity in dNK1 cells. The decreased expression of NR4A2 in RPL patients is noteworthy, as NR4A2 has been reported to regulate the expression of immune checkpoint molecules, such as TIM-3 and PD-1 [47].

One limitation of our study was the lack of matched scRNA-seq data for the ST data of RPL patients. Given the largely unknown pathogenesis of RPL and the heterogeneity observed among RPL patients, the status and signature of immune cells, such as dNK cells, may vary among individuals. A larger cohort of RPL patients and matched spatial multi-omics data with higher resolution are warranted to further elucidate the pathogenesis of RPL and identify heterogeneous subtypes among patients. It is important to note that our study focused solely on elucidating the transcriptional regulatory program of immune cells. A recent study revealed dynamic changes in the chromatin accessibility of endometrial stromal cells throughout the menstrual cycle [48]. Considering the crucial role of dS cells in embryo implantation [49] and immune niche assembly [45], further investigation is warranted to unravel the regulatory mechanisms governing dS differentiation and IL-15 production in both healthy individuals and RPL patients.

In summary, our study delineates the spatial organization of the human decidua that ensures successful pregnancy and provides deep insights into the molecular mechanisms underlying the local accumulation and immunomodulatory capabilities of key decidual immune cells.

## Materials and methods

### Human samples

All decidual tissue samples used in this study were collected from the First Affiliated Hospital of the University of Science and Technology of China. Eighteen decidual samples from normal pregnancies were obtained from elective pregnancy terminations (six for ST and twelve for scATAC-seq). Ten decidual samples were obtained from patients with unexplained recurrent spontaneous pregnancy losses (four for ST and six for scATAC-seq), and patients with genetic or anatomical causes of abortion were excluded. The elective termination was performed via dilation and curettage. For decidual samples from RPL patients, we excluded patients exhibiting clinical symptoms of vaginal bleeding and cramps before induced abortion. All the samples were collected, placed in RPMI media on ice, and transferred immediately to the laboratory for further processing within 2 h. The clinical characteristics of the enrolled pregnant women are summarized in Table S1.

### Spatial transcriptomics

Fresh decidual tissues were washed in cold phosphate-buffered saline (PBS), laid open, embedded in OCT compound (Catalog No. 4583, Sakura Finetek, Torrance, CA) in a 15 × 15 mm^2^ cryomold, and subsequently stored at −80°C. Tissue blocks were cut into 12-μm sections and processed using the Visium Spatial Gene Expression Kit (10x Genomics) according to the manufacturer’s instructions. The decidual tissue permeabilization conditions were optimized using the Visium Spatial Tissue Optimization Kit, which was found to be ideal at 6 min. The sections were stained with hematoxylin and eosin, imaged using a TissueFAX PLUS ST microscope under 20× lens magnification, and subsequently processed for ST. The final libraries were sequenced using an Illumina NovaSeq 6000 system with a paired-end 150 bp reading strategy.

### Isolation of decidual cells

Decidual tissues were washed in cold PBS containing 100 U/ml penicillin/streptomycin (Catalog No. 15140122, Gibco, Grand Island, NY), sheared into approximately 1 mm^3^ pieces, and then digested with 1 mg/ml collagenase IV (Catalog No. C5138, Sigma, St. Louis, MO) in RPMI 1640 medium (Catalog No. SH30809.01, HyClone, Logan, UT) at 37°C for 60 min with shaking at 200 r/min. The resulting suspensions were diluted with PBS and filtered through a 40-μm mesh (Catalog No. 431750, Corning, Corning, NY) to remove tissue debris. The flow-through was centrifuged, the pellet was resuspended, and mononuclear cells were enriched using a Ficoll density gradient. The separated cells were washed with PBS and used for subsequent experiments.

### Flow cytometry

Decidual mononuclear cells were washed in cold PBS and then stained with the desired antibody (Table S5) for 30 min at 4°C. The cells were washed and stained with the dead cell marker 4′,6-diamidino-2-phenylindole (DAPI; Catalog No. 422801, BioLegend, San Diego, CA) for 5 min. The cells were subsequently loaded onto a Sony SH800S flow cytometer (Sony) for data acquisition and cell sorting. Freshly sorted decidual immune cells or specific dNK cell subsets were immediately subjected to scATAC-seq or *in vitro* cell culture experiments.

### Generation of the scATAC-seq library

The scATAC-seq protocol was performed on single cells as previously described [50]. Briefly, 50,000 viable CD45^+^ cells were sorted, subjected to Tn5 tagmentation with 5 μl of Tn5 transposase (Catalog No. TD501, Vazyme, Nanjing, China), and incubated on a thermomixer at 800 r/min at 37°C for 30 min. The reaction was then stopped, and the nuclei were stained with 1 μg/μl DRAQ7 (Catalog No. 424001, BioLegend) on ice for 5 min. The samples were transferred to a FACS tube, and single nuclei were sorted into a 384-well plate (Catalog No. 4483319, Thermo Fisher Scientific, Waltham, MA) for nuclear lysis. Then, the plate was sealed and incubated at 65°C for 15 min. Library amplification was carried out using 2× PCR Master Mix (Catalog No. M0541L, NEB, Ipswich, MA), followed by purification with a QIAquick PCR purification kit (Catalog No. 28106, Qiagen, Valencia, CA). Finally, the amplified library was sequenced using the Illumina HiSeq X Ten platform.

### dNK cell culture *in vitro*

DAPI^−^CD45^+^CD56^+^CD103^−^CD39^−^CD18^+^ dNK2 cells were sorted from healthy controls by flow cytometry and cultured with RPMI 1640 supplemented with 10% fetal bovine serum (FBS; Catalog No. 10091-148, Gibco, Auckland, New Zealand) and 100 U/ml penicillin/streptomycin in a 96-well U-bottom plate (Catalog No. 10360691, Corning). Subsequently, 10 ng/μl human recombinant IL-15 (Catalog No. AF-200-15, PeproTech, Rocky Hill, NJ) was added to induce the transformation of dNK2 cells, and the control group was supplemented with PBS only. After 48 h, the cultured cells were collected and stained with DAPI, and the following antibodies were used: PerCP/Cyanine5.5 anti-human CD45 (Catalog No. 304028, BioLegend), Alexa Fluor® 647 anti-human CD56 (Catalog No. 362514, BioLegend), PE anti-human CD18 (Catalog No. 302107, BioLegend), FITC anti-human CD39 (Catalog No. 328205, BioLegend), and PE/Cyanine7 anti-human CD103 (Catalog No. 350211, BioLegend). The expression of CD39 was then detected using flow cytometry, and DAPI^−^CD45^+^CD56^+^ dNK cells after IL-15 or PBS stimulation were sorted for RNA-seq experiments.

### RNA-seq

The desired live dNK cells were sorted by flow cytometry, and up to 1000 cells were collected directly in a 0.2-ml PCR tube (Catalog No. KG2511, KIRGEN Bioscience, Shanghai, China). The RNA-seq library was constructed following the established protocols of Smart-seq2 [51]. The resulting libraries were sequenced on the Nova-seq 6000 platform, with an average of 20 million paired reads generated per sample.

### CUT&Tag

The CUT&Tag assay was performed using the Hyperactive In Situ ChIP Library Kit for Illumina (Catalog No. TD904, Vazyme) according to the manufacturer’s recommendation. Briefly, 5 × 10^4^ dNK1 cells from healthy controls were sorted, incubated with concanavalin A-coated magnetic beads and incubated with a primary antibody (rabbit anti-FOSL2; Catalog No. 19967, Cell Signaling Technology, Shanghai, China) overnight at 4°C. No primary antibody was added to the negative control group. Then, the secondary anti-rabbit antibody (goat-anti-rabbit; Catalog No. ab6702, Abcam, Cambridge, UK) was added, after which the cells were washed and incubated with the pG-Tn5/pA-Tn5 transposon (Catalog No. TD904, Vazyme) for 1 h at room temperature. The extracted DNA fragments were sequenced on an Illumina NovaSeq 6000 system.

### Lentivirus transduction of dNK cells

*FOSL2*-KD cells were generated via transduction of shRNA-expressing lentivirus (GenePharma). The sequences of the shRNAs used to target *FOSL2* are listed in Table S6. For lentivirus transduction, 1 × 10^5^ dNK2 cells were sorted and cultured in a 96-well U-bottom plate containing RPMI 1640 supplemented with 10% FBS and 100 U/ml penicillin/streptomycin. The cells were mixed with the lentivirus [multiplicity of infection (MOI) = 30] in the presence of 5 μg/μl polybrene (Catalog No. TR-1003-G, Sigma) in a final volume of 200 μl. The plate was then incubated at 37°C with 5% CO_2_ for 6 h, followed by centrifugation at 500 *g* for 5 min. Subsequently, the supernatant was removed and replaced with 200 μl of fresh RPMI 1640 supplemented with 10% FBS and 100 U/ml penicillin/streptomycin. Additionally, 1 ng/μl of IL-15 was added during lentivirus transduction to maintain the survival of NK cells. After 2 days of incubation, the culture medium was refreshed, and 10 ng/μl IL-15 was added to stimulate the dNK cells. After 2 days of stimulation, the expression of CD39 was analyzed using flow cytometry, and DAPI^−^GFP^+^CD56^+^ dNK cells were sorted for subsequent RT-qPCR and RNA-seq experiments.

### RT-qPCR

The expression of selected candidate genes was validated by RT-qPCR. Briefly, cDNA was synthesized with Maxima H Minus Reverse Transcriptase (Catalog No. EP0751, Thermo Fisher Scientific). Then, KAPA SYBR FAST qPCR (Catalog No. KK4600, Sigma) was used for RT-qPCR in accordance with the manufacturer’s instructions. The data were collected using a LightCycler96 fluorescent sequence detection system and analyzed using the 2^−ΔΔCT^ method, and the statistical significance of the results was assessed using a paired Student’s *t*-test. The sequences of primers used for RT-qPCR are listed in Table S6.

### Visium ST data processing

The reads were demultiplexed and mapped to the human reference genome GRCh38 using Space Ranger software (v.1.2.1; 10x Genomics). StarDist [18] was used to process the histological images of the 10x Visium data and calculate the number of cells per spot. After eliminating ST spots with a cell number ≤ 0, count matrices were loaded into Scanpy (v.1.9.1; https://scanpy.readthedocs.io/en/stable/) for data normalization and log transformation.

### Cell type deconvolution and spatial domain identification

To estimate the cell type proportions of each spot in the ST data, we first downloaded publicly available human decidua scRNA-seq datasets from healthy controls [12] (ArrayExpress database, E-MTAB-6701) and RPL patients [4,6] (GSA database, CRA002181). Cell2location (v0.6a0) [19] was subsequently used to deconvolve the cell type composition of each ST spot by integrating the scRNA-seq and ST data from healthy controls and RPL patients using the following hyperparameters: N_cells_per_location = 30 and detection_alpha = 200. For each cell type, the original distribution probability provided by Cell2location was normalized by the proportion of the cell type in the single-cell data, resulting in the normalized distribution probability. The distribution probabilities of 17 cell types were utilized as inputs for spatial domain identification using SPACEL [20]. The domain signature genes and the genes differentially expressed between healthy donors and RPL patients were identified using Seurat’s FindAllMarkers function (v4.2.0) [52], with thresholds set at |log_2_ FC| ≥ 0.5 and *P* < 1 × 10^−10^. The Jaccard index between domain signature genes was calculated to measure the transcriptome similarity of spatial domains. The cell type distribution results were further confirmed using an alternative deconvolution method, Tangram (v1.0.4) (mode = cluster) [24].

### Functional annotation

GO enrichment analysis of the query gene set was performed using the online tool Metascape (https://metascape.org/). *P* values were calculated using a one-sided hypergeometric test.

### Cellular colocalization analysis

For cellular colocalization analysis, we computed the Pearson correlation coefficient for pairwise cell type distribution probabilities within each ST section. The five coefficients with the highest values were retained for each cell type, and the remaining coefficients were set to zero. The correlation matrices of the ST sections from healthy donors or RPL patients were separately averaged to generate the cell type colocalization correlation matrix.

### Cell–cell interaction analysis

A distance-smoothed expression matrix was first computed by averaging the expression of a given spot and its directly adjacent spots. Then, for each LR pair in the CellPhoneDB resource [12], we calculated the Pearson correlation coefficient between the smoothed expression of the receptor and ligand within each slide. LR pairs with correlation coefficient ≥ 0 and *P* < 0.05 were considered spatially coexpressed LR pairs, and the expression value of a specific LR pair in each spot was determined as the product of the smoothed receptor and ligand expression. LR pairs that exhibited upregulation in a specific spatial domain or in normal/disease states were identified through differential expression analysis (log_2_ FC ≥ 0.1 and *P* < 1 × 10^−5^) using the FindAllMarkers function in Seurat. *P* values were corrected for multiple testing using the false discovery rate method. For each LR pair, we assigned the cell type pair that was colocalized (the Pearson correlation coefficient of the probability distribution between two cell types ≥ 0.1) and had the highest expression to the LR pair. CellChat (v1.4.0) [53] was used for functional pathway annotation of the differentially expressed LR pairs.

### Trajectory analysis for dNK cell transformation

Tangram [24] was used to determine the spatial location of individual dNK cells by integrating scRNA-seq and ST data. We selectively retained cells that were mapped to cell type-enriched spots for each dNK cell subset. A spot was considered cell type-enriched if the cell type distribution probability was greater than or equal to the average distribution probability (and not less than 0.1). RNA velocity analysis was conducted using scVelo (v0.2.4) [27] to examine the trajectory of the dNK cells. The transition confidence between dNK cell subsets was calculated by the scv.tl.paga function in scVelo. For further trajectory analysis, veloVI (v0.3.1) [28], a deep generative model that estimates RNA velocity uncertainty through posterior sampling, was employed. A total of 100 velocity fields were generated from the posterior distribution for transition confidence evaluation. To assess the statistical significance of the observed transformation trajectories, we performed a permutation test by randomly shuffling cell-type labels 1000 times and recalculating the transition confidence for each iteration. Empirical *P* values were determined by comparing the observed transition confidence with the null distribution of random values generated from label shuffling.

### Upstream ligand analysis for dNK cell transformation

To explore the key molecules that regulate the transformation of dNK cells, genes that were positively correlated with the distribution of dNK1 cells were identified for upstream ligand prediction. Specifically, the relationship between the expression of each dNK cell signature gene and the probability of distribution of dNK1 cells was evaluated using the maximal information coefficient (MIC). Genes with MIC values ≥ 0.9 were selected for subsequent spatial expression clustering analysis, which identified 204 genes whose expression was positively correlated with the dNK1 cell distribution probability. These genes were subsequently utilized as downstream target genes in the NicheNet package (v1.1.0) [30] for upstream ligand prediction.

### Acquisition of gene sets

The gene sets associated with specific dNK cell functions used in this study were collected (Table S4). Furthermore, gene sets related to a specific GO term were obtained from MSigDB (https://www.gsea-msigdb.org/gsea/msigdb).

### scATAC-seq data analysis

The raw fastq data were preprocessed using APEC software (v1.1.0; https://github.com/QuKunLab/APEC) [54]. After trimming adapter sequences the reads were mapped to the hg38 genome. Subsequently, PCR duplicates were removed. The uniquely mapped reads were shifted, extended to 50 bp centered through the cleavage position, and then merged for peak calling. Cells with ≥ 1200 unique fragments and a fraction of reads in peaks ≥ 0.2 were retained for further analysis. Clustering analysis was performed using the ‘clustering.cluster_byAccesson’ function in APEC with default parameters. TF binding activity was computed using the ‘clustering.cluster_byMotif’ function, and TF binding activity per spot was calculated by summing the product of the distribution probability and TF binding activity of the three dNK cell subsets. For dNK cell pseudotime trajectory inference, we used the ‘addIterativeLSI’ function of the ArchR package (v0.9.5) [55] to perform iterative latent semantic indexing (LSI). The pseudotime trajectory inference of dNK cells was inferred using the Palantir package (v1.0.0) [31], which took the LSI matrix as input. TF footprinting analysis was conducted using HINT-ATAC [56].

### CUT&Tag data analysis

The raw reads were trimmed to 50 bp, and adapter sequences were removed. The trimmed sequences were aligned to hg38 using Bowtie2 with the following options: --local --very-sensitive --no-mixed --no-discordant --phred33 -I 10 -X 700. Low-quality reads with a MAPQ < 2 were filtered out. MACS2 [57] was employed for peak calling utilizing the following parameters: -t target_file -c igg_file --keep-dup all --nomodel --shift 0. A *P* value threshold of 1 × 10^−35^ was applied to select high-quality peaks. The IDR algorithm provided by the ENCODE project (v2.0.4.2; https://www.encodeproject.org/software/idr) was used to identify reproducible binding sites between two biological replicates. Genomic regions were annotated using the “annotateOpenregions.pl” function of HOMER software (http://homer.ucsd.edu/homer/motif/).

### RNA-seq data analysis

The raw data were aligned to hg38 using STAR. Gene expression was quantified using HTSeq (v0.13.5; https://htseq.readthedocs.io/en/master/). The DESeq2 R package (v1.24) was used to normalize read counts and compute DEGs. A *P* value < 0.05 and |log_2_ FC| ≥ 0.5 (for the IL-15 stimulation experiment) or ≥ 0.2 (for the shRNA experiment) were considered to indicate statistical significance.

### Signaling network construction

We selected FOSL2 target genes (by surveying CUT&Tag data) and IL-15 signaling genes to construct a signaling network that focuses on the regulation of immunoregulatory functions (by combining the “Immunoregulatory” gene set from Table S4 and a gene set related to “negative regulation of immune system process” from https://www.gsea-msigdb.org/gsea/msigdb). In the network, the edges represent either regulation by FOSL2 or upregulation by IL-15, while the nodes represent genes targeted by FOSL2 and/or downstream of IL-15 signaling. Downstream genes of the IL-15 signaling pathway were defined as genes upregulated by IL-15 stimulation and/or belonging to the “REACTOME_INTERLEUKIN_15_SIGNALING” term in the MSigDB database. Each node was assigned a color corresponding to the type of differential expression exhibited by the gene, and the shape of each node indicates the type of gene regulation.

### Simulated *FOSL2* overexpression analysis

*FOSL2* overexpression was simulated in dNK cells from RPL patients using SCENIC+ [42]. We began by aligning the scRNA-seq and scATAC-seq data (using nr_cells_per_metacell = 2) and constructing a gene regulatory network with the build_grn function. Subsequently, *FOSL2* overexpression was simulated to perturb the transcriptional activity of the dNK cells, and the resulting gene expression data were analyzed to identify differentially regulated genes following the simulated overexpression. Trajectory analysis was performed to investigate the transformation from dNK2 to dNK1-like states following simulated *FOSL2* overexpression. To further explore the impact of *FOSL2* expression levels, dNK2 cells from RPL patients were stratified into two groups based on *FOSL2* expression, using the 25th and 75th percentiles of *FOSL2* expression as cutoffs. Cells with *FOSL2* expression below the 25th percentile were categorized as *FOSL2*-low, while cells above the 75th percentile were classified as *FOSL2*-high. Differential gene expression between these groups was assessed, focusing on dNK1 signature genes and immunoregulatory genes.

### Statistical analysis

Statistical significance was assessed using a two-sided Wilcoxon rank-sum test, chi-square test, two-sided Student’s *t*-test, paired Student’s *t*-test, or two-sided ANCOVA with gestational age as a covariate, using Python or Prism software (GraphPad). Sample sizes were not predetermined using statistical methods. Statistical significance is represented by *, *P* < 0.05; **, *P* < 0.01; ***, *P* < 0.001; ****, *P* < 0.0001.

## Ethical statement

This study was approved by the Ethics Committee of the First Affiliated Hospital of the University of Science and Technology of China (Approval No. 2020KY Trial No. 16), and written informed consent was obtained from all participants. Research involving patients enrolled in this study was conducted in accordance with the principles of the Declaration of Helsinki.

## Code availability

The source code is available at GitHub (https://github.com/QuKunLab/Decidua). The code has also been submitted to BioCode at the National Genomics Data Center (NGDC), China National Center for Bioinformation (CNCB) (BioCode: BT007938), which is publicly accessible at https://ngdc.cncb.ac.cn/biocode/tool/BT007938.

## Supplementary Material

qzaf080_Supplementary_Data

## Data Availability

The raw sequencing data have been deposited in the Genome Sequence Archive for Human [58] at the NGDC, CNCB (GSA-Human: HRA003056), and are publicly accessible at https://ngdc.cncb.ac.cn/gsa-human.

## CRediT author statement

**Qing Sha:** Validation, Investigation, Visualization, Methodology, Writing – original draft, Writing – review & editing. **Qiaoni Yu:** Software, Data curation, Formal analysis, Investigation, Visualization, Methodology, Writing – original draft, Writing – review & editing. **Kaixing Chen:** Software, Formal analysis. **Junyu Wang:** Software, Formal analysis. **Feiyang Wang:** Writing – review & editing. **Chen Jiang:** Formal analysis. **Yuanzhe Li:** Formal analysis. **Meifang Tang:** Validation. **Yanbing Hou:** Validation. **Ke Liu:** Validation. **Kun Chen:** Formal analysis. **Zongcheng Yang:** Writing – review & editing. **Shouzhen Li:** Writing – review & editing. **Jingwen Fang:** Writing – review & editing. **Sihui Luo:** Resources. **Xueying Zheng:** Resources. **Jianping Weng:** Resources. **Kun Qu:** Conceptualization, Resources, Funding acquisition, Writing – review & editing, Supervision, Project administration. **Chuang Guo:** Conceptualization, Resources, Funding acquisition, Writing – review & editing, Supervision, Project administration. All authors have read and approved the final manuscript.

## ORCID

0000-0002-8604-1975 (Qing Sha)

0000-0003-3272-7119 (Qiaoni Yu)

0009-0000-8485-1432 (Kaixing Chen)

0009-0000-0621-3435 (Junyu Wang)

0000-0002-7866-5497 (Feiyang Wang)

0000-0002-1415-185X (Chen Jiang)

0009-0002-1474-2809 (Yuanzhe Li)

0009-0002-9780-7232 (Meifang Tang)

0009-0006-6028-7578 (Yanbing Hou)

0000-0001-9754-1492 (Ke Liu)

0009-0007-0147-651X (Kun Chen)

0000-0001-8272-3728 (Zongcheng Yang)

0000-0002-3780-5190 (Shouzhen Li)

0000-0001-7906-8152 (Jingwen Fang)

0000-0001-8503-0310 (Sihui Luo)

0000-0001-8395-9476 (Xueying Zheng)

0000-0002-7889-1697 (Jianping Weng)

0000-0002-5555-8437 (Kun Qu)

0000-0002-3912-0793 (Chuang Guo)
