# Supplementary material for: Spatial Transcriptomics of Human Decidua Identifies Molecular Signatures in Recurrent Pregnancy Loss
Source: Genomics Proteomics Bioinformatics. 2025 Oct 1;24(1):qzaf080. doi: 10.1093/gpbjnl/qzaf080 (PMC13242933; doi:10.1093/gpbjnl/qzaf080)
Supplement: qzaf080_Supplementary_Data [file qzaf080_supplementary_data.zip › Supplementary material captions.docx]

**Supplementary material**

**Figure S1 Spatial domain identification in the deciduas from healthy donors and RPL patients**

**A.** Spatial domains (IZ and GZ) identified in the deciduas from healthy donors (N59R2, N56R1, N56R2, and N65R2) and RPL patients (P4 and P9R2). The top panel shows the corresponding histology image. Scale bar, 1 mm. **B.** Spatial expression of known layer-specific genes (decidua compacta: *PRL*, *CYP11A1*, and *IGFBP2*; decidua spongiosa: *ACTA2* and *TAGLN*) in the deciduas of healthy donors and RPL patients. **C.** Spatial distribution proportions of dS1, dS2, and dS3 cells in the decidua of healthy donors and RPL patients. **D.** Functional annotation of IZ and GZ signature genes.

**Figure S2 Comparison of cell type deconvolution results between Cell2location and Tangram**

**A.** Comparison of cell type distribution in IZ and GZ of healthy controls and RPL patients. **B.** Normalized distribution probabilities (proportions) of the 17 cell types in IZ and GZ given by the two methods. The original distribution probability for each cell type was normalized by the proportion of the cell type in the single-cell data, resulting in the normalized distribution probability. **C.** Spatial distribution probabilities of dS2, dNK1, dM2, and CD8^+^ T cells in the decidua given by the two methods. **D.** Within-spot Pearson correlation analysis of cell type distribution probabilities by the two methods.

**Figure S3 Differentially expressed LR pairs between RPL patients and healthy controls in IZ and GZ of the decidua**

**A.** Pie chart displaying the proportion of coexpressed LR pairs upregulated in a specific domain (IZ or GZ) for each sample. **B.** Bar plot showing the number of LR pairs upregulated in spatial domains of healthy donors and RPL patients, respectively. **C.** Summary of specific signaling pathways as well as annotated LR pairs and cell type pairs. **D.** Volcano plot showing differentially expressed LR pairs in IZ (left) and GZ (right) between RPL patients and healthy controls. LR pairs related to collagen signaling are highlighted in orange, while other upregulated LR pairs in RPL (blue) and controls (yellow) are also shown. The red dashed line indicates an adjusted *P* value = 1×10^-5^. **E.** Box plot comparing the log_2_ FC of LR pairs upregulated in IZ of healthy controls, with a focus on collagen-related LR pairs (orange) versus others (blue). Statistical significance was determined by a two-sided Student’s *t*-test (*, *P* < 0.05).

**Figure S4 Cell‒cell interactions in spatial domains of healthy donors and RPL patients**

**A.** Box plot showing the Pearson correlation coefficient of expression between each collagen gene and inhibitory NKRs in the deciduas of healthy donors and RPL patients. Statistical significance was determined by a two-sided Wilcoxon rank-sum test (*, *P* < 0.05). **B.** and **C.** Spatial average expression of LR pairs associated with the CSF (B) or CD39 (C) signaling pathway in the decidua of healthy donors and RPL patients. Statistical significance of the LR average expression was determined by two-sided ANCOVA (***, *P* < 0.001). **D.** Box plot showing the expression of *CXCL14* (left) and *CXCR4* (right) in cell types from healthy donors and RPL patients. Statistical significance was determined by a two-sided Student’s *t*-test (****, *P* < 0.0001). **E.** Spatial distribution probability of Epi, dS2, and CD8^+^ T cells, as well as spatial average expression of LR pairs associated with the CXCL pathway in RPL patients around glandular regions, as shown in the histology image. Epi, Epithelial.

**Figure S5 Spatial transformation of dNK2 cells to dNK1 cells in the compacta layer**

**A.** RNA-velocity stream of dNK cells at spatial resolution. **B.** Heatmap showing the transition confidence between dNK cell subsets in specific domains using PAGA. **C.** Box plot showing the transition confidence from dNK2 cells (IZ) to dNK1 cells (IZ) in the deciduas from healthy donors and RPL patients. Statistical significance was determined by a two-sided Wilcoxon rank-sum test (*, *P* < 0.05). **D.** Box plot comparing 100 velocity field trajectories generated by veloVI through posterior probability sampling. Each point is the average of the transition confidence of the trajectory generated by a sampling in healthy donors or RPL patients. Statistical significance was determined by a two-sided Student’s *t*-test (****, *P* < 0.0001). **E.** Schematic diagram illustrating the permutation test workflow. The observed trajectory (left) is compared to randomized trajectories generated by label shuffling (right). The process is repeated 1000 times to calculate random transition confidence and derive empirical *P* values for each trajectory. **F.** Bar plot showing the statistical significance of observed transition confidence, computed via 1000 permutation tests. The y-axis represents the negative log_10_ of the *P* value for each transition, with the dashed lines indicating significance thresholds of *P* = 0.05 and *P* = 0.01. PAGA, Partition-based graph abstraction.

**Figure S6 Association of IL-15 signaling with dNK cell subsets**

**A.** ViolinPlot showing the expression of genes encoding IL-15 receptors in dNK cell subsets from healthy donors and RPL patients. **B.** and **C.** Box plot showing the Pearson correlation of dNK cell distribution probability and *IL15* (B) or *IL2RB* (C) expression in healthy donors vs RPL patients. Statistical significance was determined by a two-sided Wilcoxon rank-sum test (**P* < 0.05). **D.** Bar plot showing the enrichment of IL-15 upregulated genes associated with dNK1 cell distribution in terms of known dNK functions.

**Figure S7 QC of scATAC-seq data**

**A.** and **B.** TSS enrichment scores (A) and fragment length distribution (B) of the scATAC-seq data. **C.** QC assessment of scATAC-seq data, with the dotted line indicating the QC screening criteria used in this study. **D.** UMAP visualization of cell type annotation and batch information in scATAC-seq data. **E.** Genome tracking plots showing genomic peaks of canonical cell type markers in the scATAC-seq data. **F**. Heatmap showing the Jaccard similarity between the unsupervised clustering result and the label transfer result generated by Seurat. QC, quality control; TSS, Transcription start site; UMAP, uniform manifold approximation and projection.

**Figure S8 Regulatory mechanism of FOSL2 in dNK1 cells.**

**A.** UMAP plot of pseudotime for dNK cells. **B.** Functional annotation of FOSL2-targeted dNK1 signature genes.

**Figure S9** **Loss-of-function experiments revealed impaired dNK cell transformation and function following FOSL2 KD**

**A.** Schematic diagram illustrating the shRNA-mediated knockdown experiment. **B.** Gating strategy for cultured cells successfully transduced with shRNA lentivirus, as detected by GFP expression. **C.** Box plot showing the relative expression of FOSL2 determined via RT-qPCR. The statistical significance was determined by a paired Student’s *t*-test (**, *P* < 0.01). **D.** Bar plot showing the number of genes differentially expressed after FOSL2 KD. The pie chart indicates the proportion of differentially expressed genes bound by FOSL2. **E.** Functional annotation of FOSL2 target downregulated genes after FOSL2 KD. **F.** Venn diagram showing the overlap between FOSL2 target downregulated genes after FOSL2 KD and genes associated with negative regulation of immune system processes. The statistical significance was determined by the chi-squared test. **G.** Box plot showing the mean expression of IL-15 upregulated genes in control and shFOSL2 cells. KD, knockdown; shRNA, short hairpin RNA; RT-qPCR, real-time quantitative PCR.

**Figure S10 Impact of *FOSL2* overexpression on dNK cell differentiation and gene expression**

**A.** Box plot showing the average expression of dNK1 signature genes before and after the simulated *FOSL2* overexpression. Statistical significance was determined by a two-sided Student’s *t*-test (****, *P* < 0.0001). **B.** Bar plot comparing the expression of individual genes before and after the simulated FOSL2 overexpression. Statistical significance was determined by a two-sided Student’s *t*-test (***, *P* < 0.001, ****, *P* < 0.0001). **C.** UMAP plots showing the difference in dNK cell transformation trajectory before (left) and after (right) *FOSL2* overexpression simulation. **D.** Density plot showing the distribution of *FOSL2* expression in dNK2 cells from RPL patients (*n* = 1177). The 25th and 75th percentiles of *FOSL2* expression are marked, dividing the cells into two groups: *FOSL2*-low (blue) and *FOSL2*-high (orange). **E.** Box plot showing the average expression of dNK1 signature genes targeted by FOSL2 in the *FOSL2*-low and *FOSL2*-high groups. Statistical significance was determined by a two-sided Student’s *t*-test (**, *P* < 0.01). **F.** Bar plot comparing the expression of individual genes regulated by FOSL2 in the *FOSL2*-low and *FOSL2*-high groups. Statistical significance was determined by a two-sided Student’s *t*-test (*, *P* < 0.05; **, *P* < 0.01).

**Table S1 Clinical characteristics of healthy controls and RPL patients included in this study**

**Table S2 Data quality of decidual samples used in ST analysis**

**Table S3 The Z-scaled matrix of differentially expressed genes in each spatial domain**

**Table S4 Collection of genes known to be related to dNK cell functions**

**Table S5 Antibodies used in this study**

**Table S6 Primer sequences for RT-qPCR and shRNA oligo sequences**
