# Supplementary figures and images for: Spatial Transcriptomics of Human Decidua Identifies Molecular Signatures in Recurrent Pregnancy Loss
Source: Genomics Proteomics Bioinformatics. 2025 Oct 1;24(1):qzaf080. doi: 10.1093/gpbjnl/qzaf080 (PMC13242933; doi:10.1093/gpbjnl/qzaf080)

**A**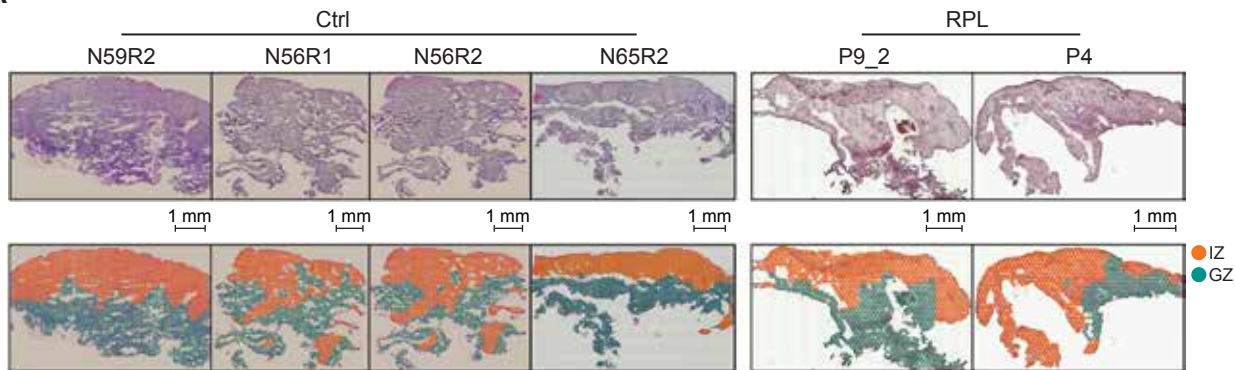**B**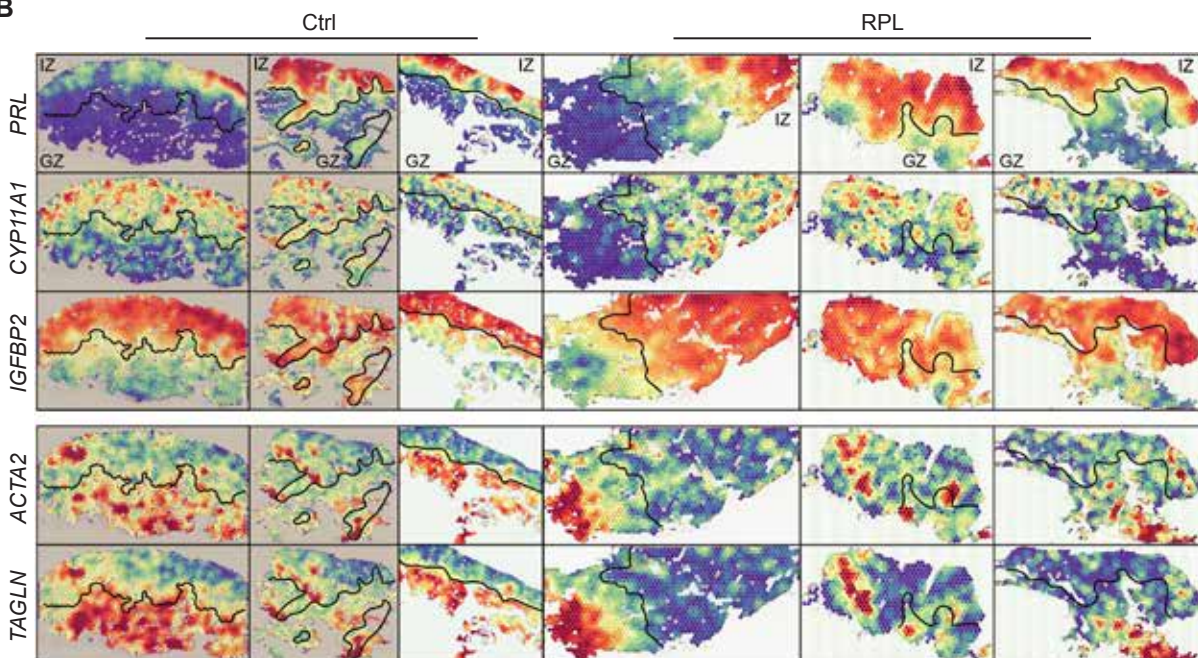**C**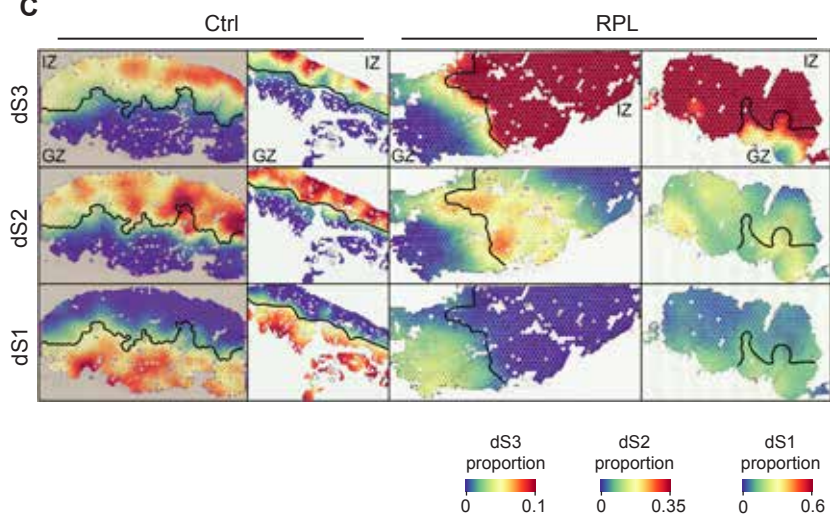**D**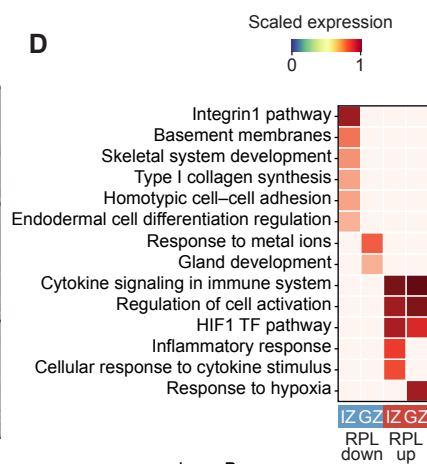

Supplement: qzaf080_Supplementary_Data [file qzaf080_supplementary_data.zip › Figure S1.pdf]

**A**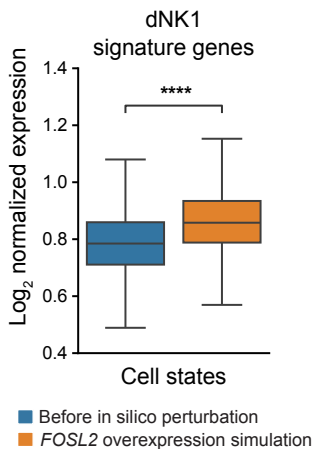**B**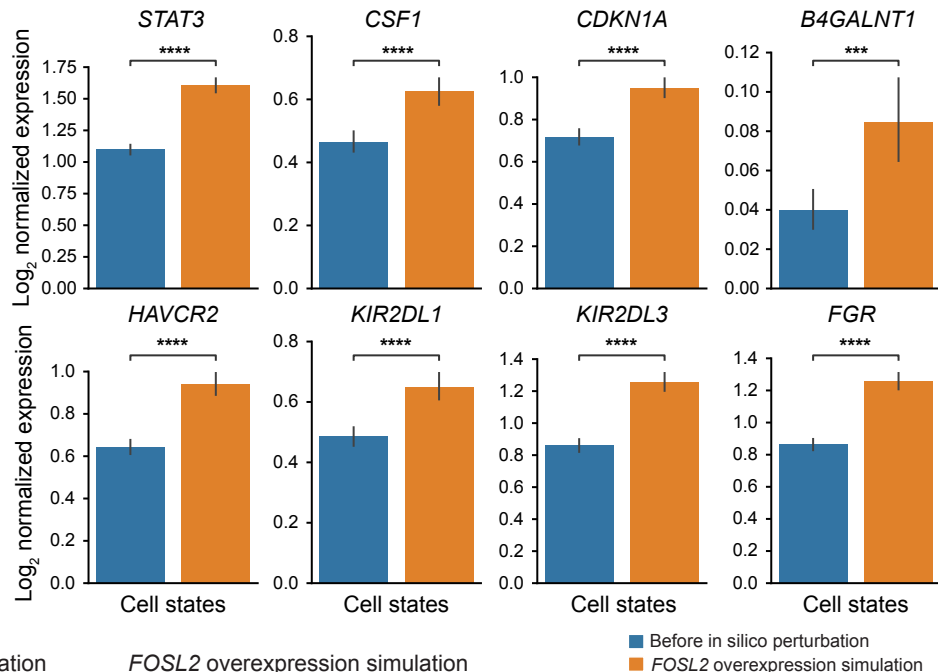**C**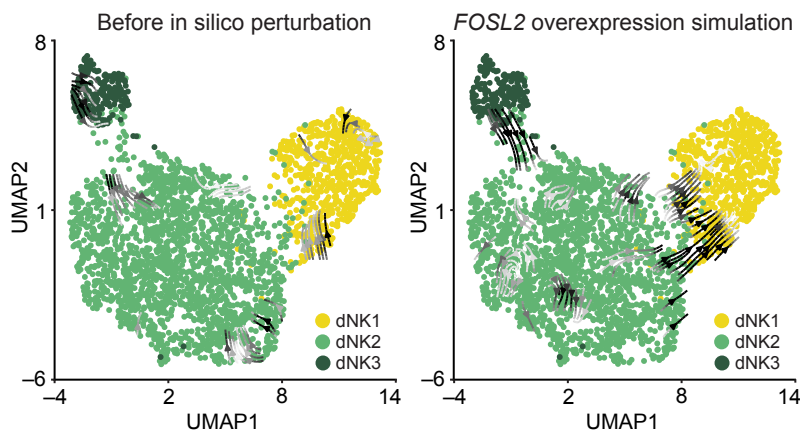**D**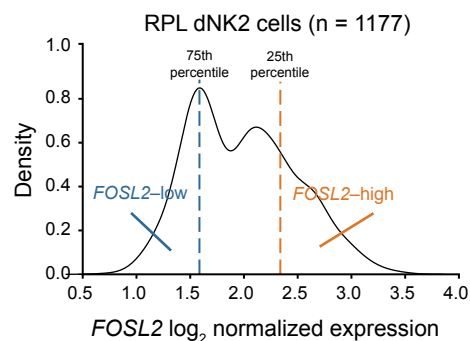**E**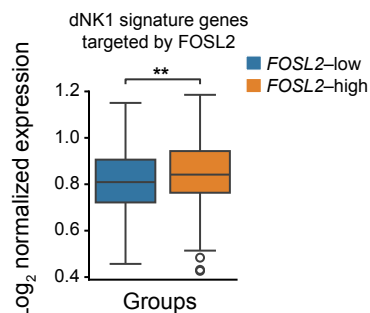**F**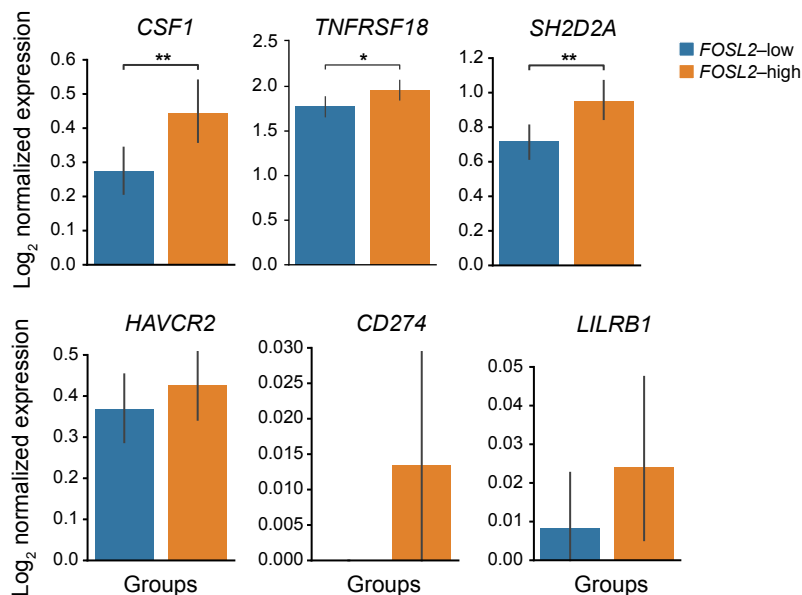

Supplement: qzaf080_Supplementary_Data [file qzaf080_supplementary_data.zip › Figure S10.pdf]

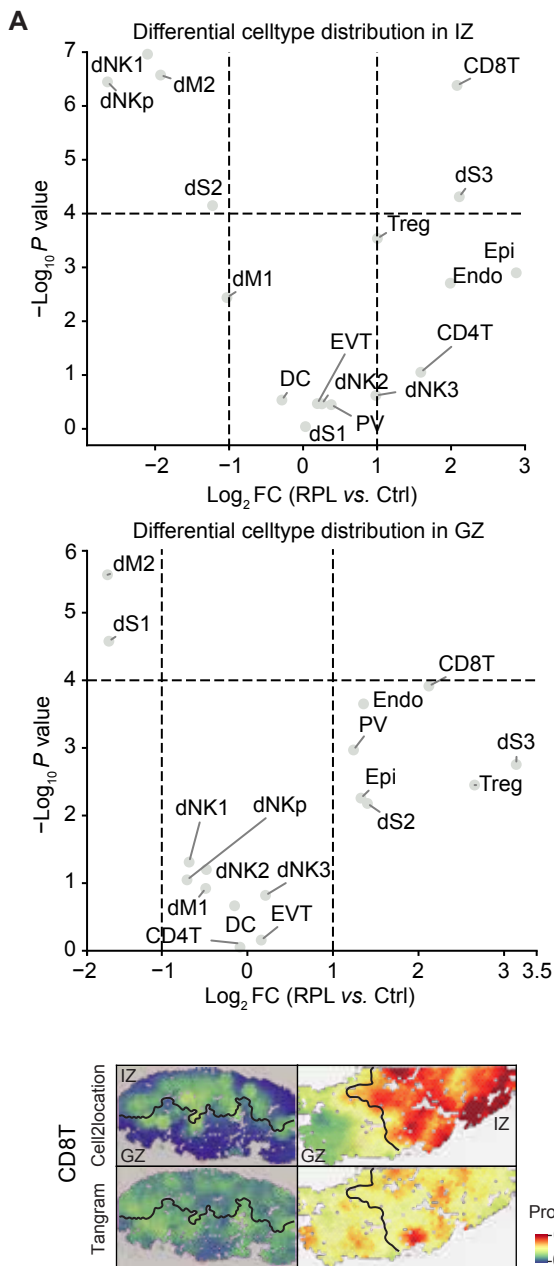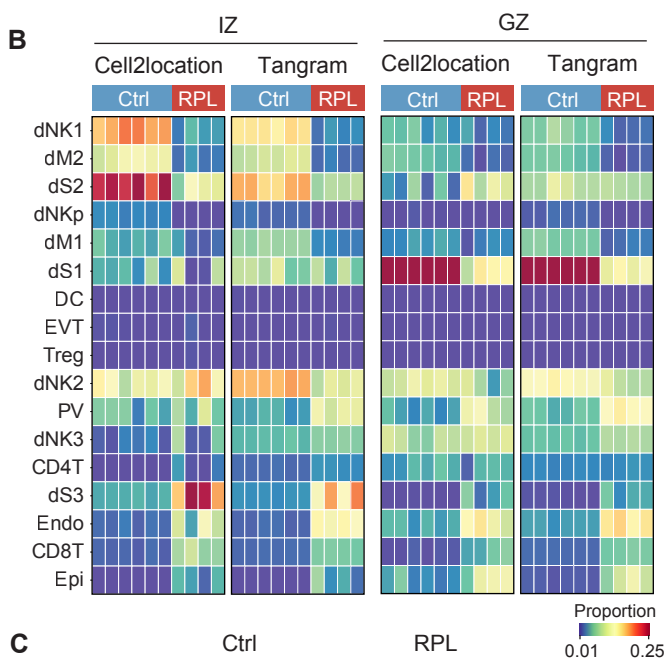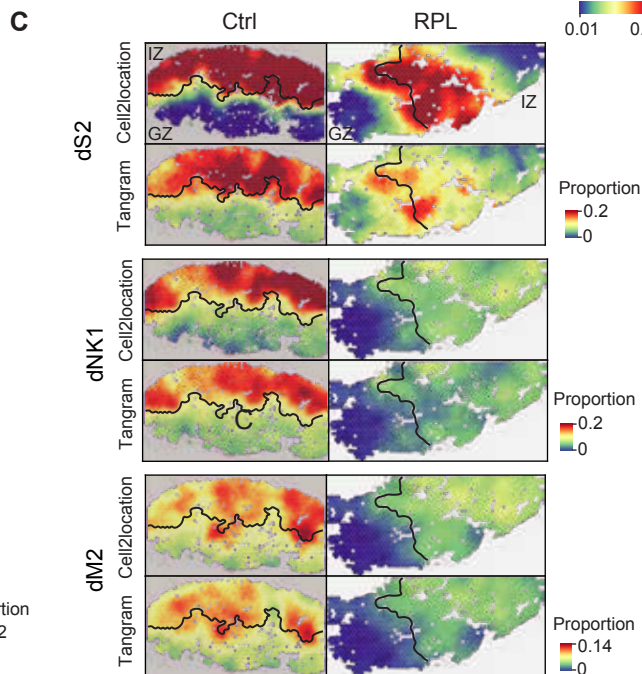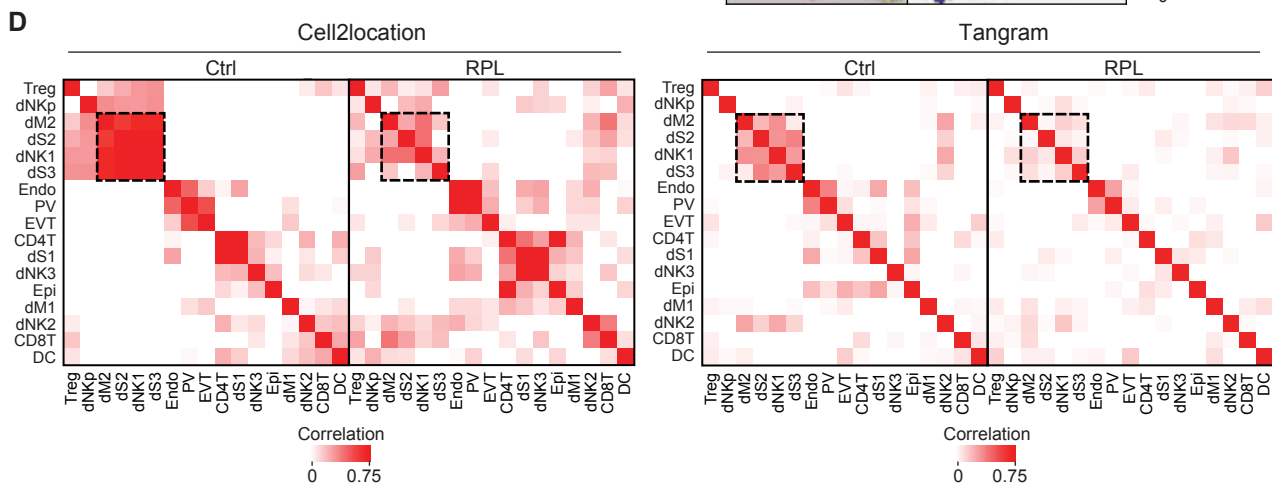

Supplement: qzaf080_Supplementary_Data [file qzaf080_supplementary_data.zip › Figure S2.pdf]

A

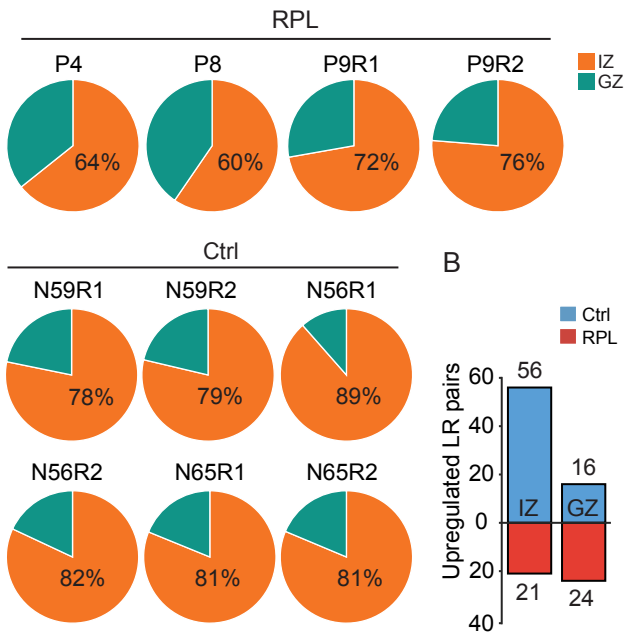

C

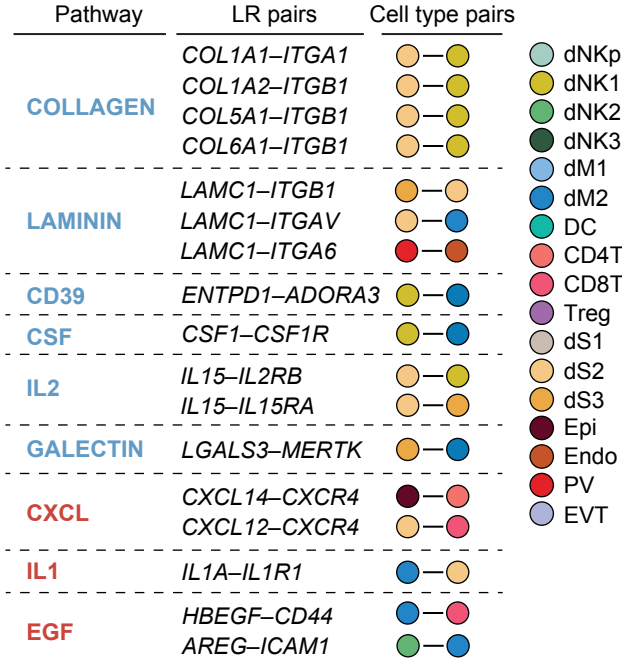

D

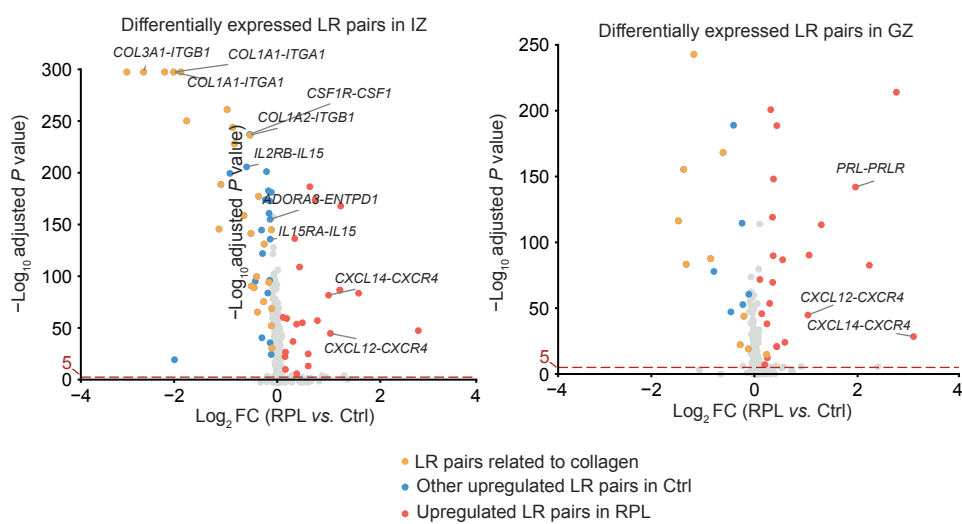

E

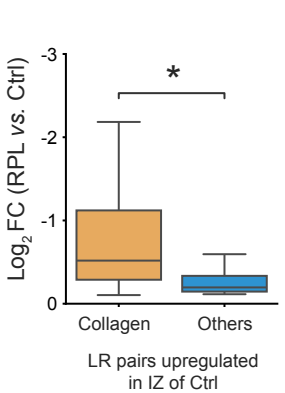

Supplement: qzaf080_Supplementary_Data [file qzaf080_supplementary_data.zip › Figure S3.pdf]

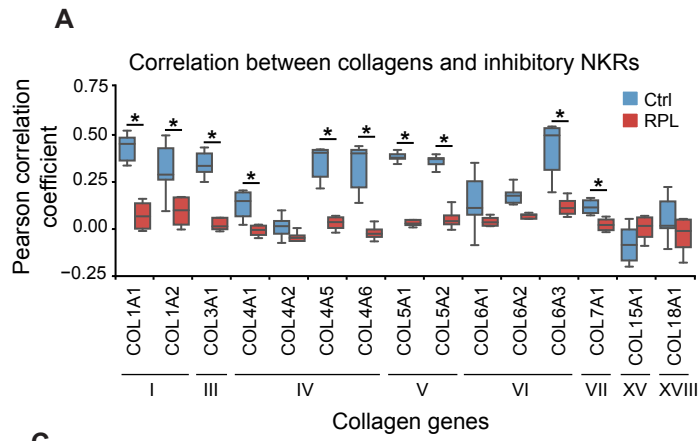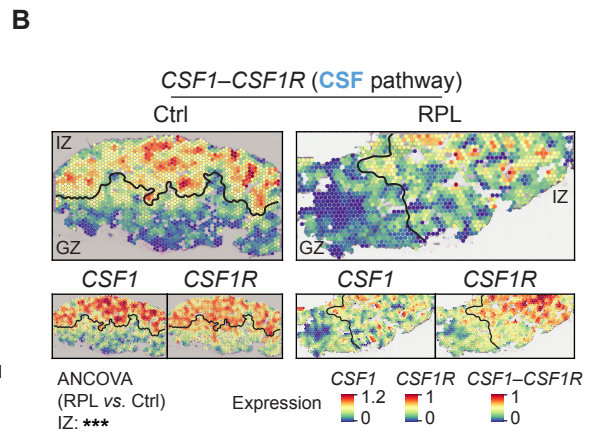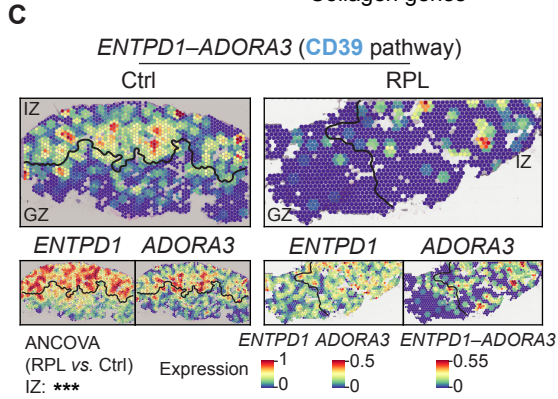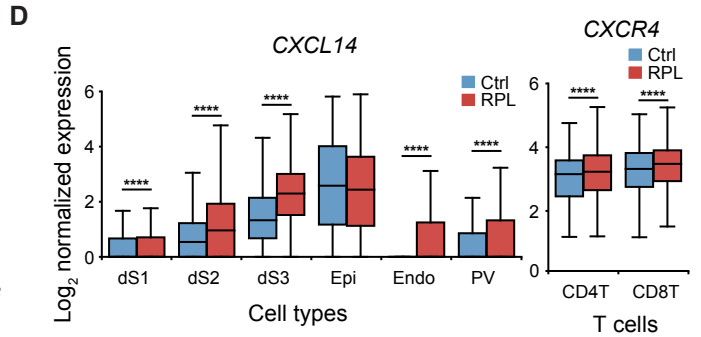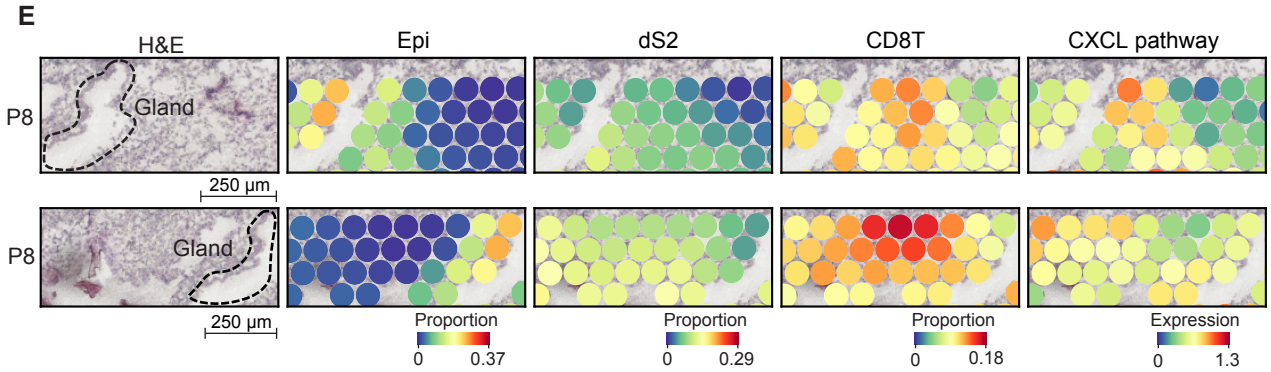

Supplement: qzaf080_Supplementary_Data [file qzaf080_supplementary_data.zip › Figure S4.pdf]

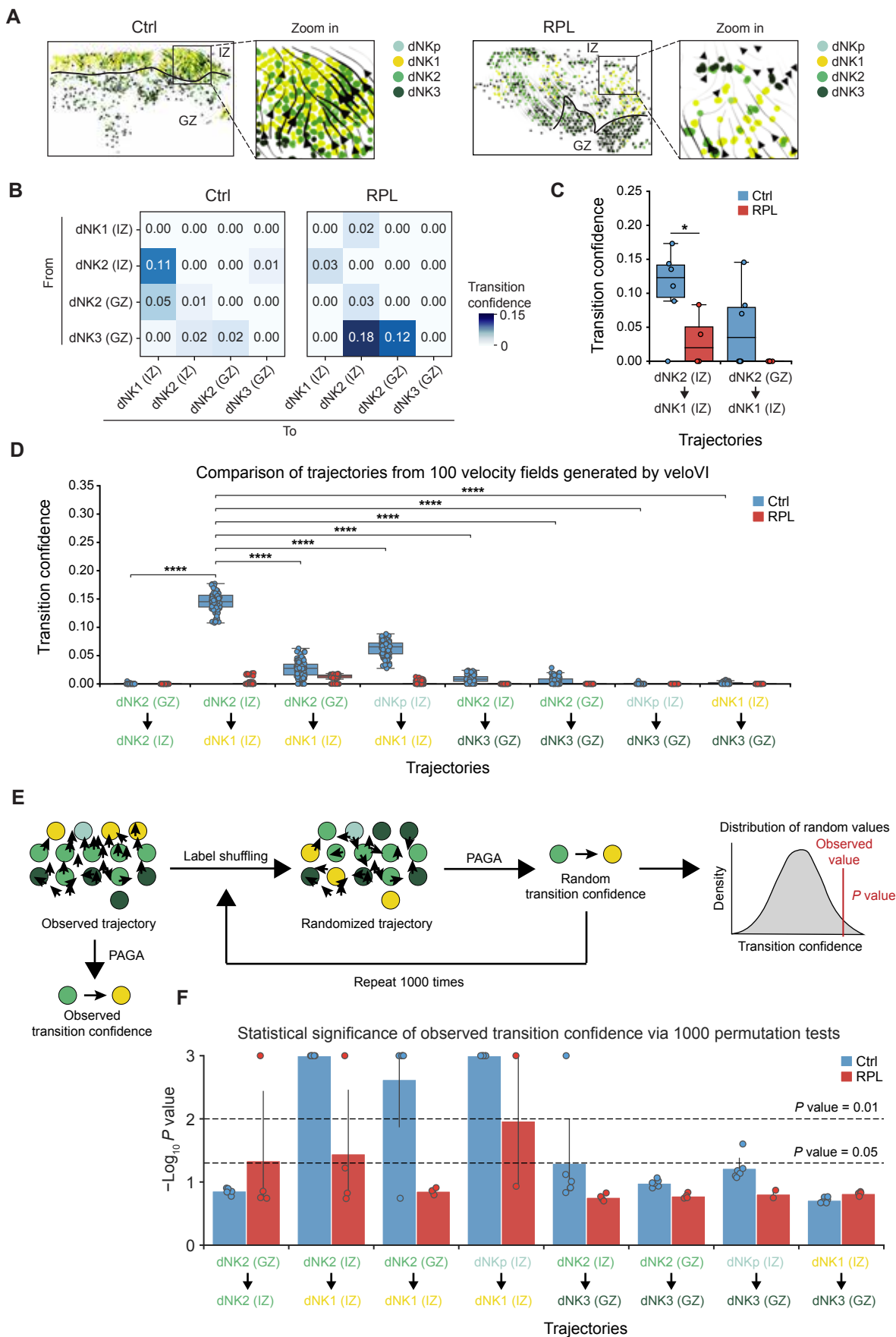

Supplement: qzaf080_Supplementary_Data [file qzaf080_supplementary_data.zip › Figure S5.pdf]

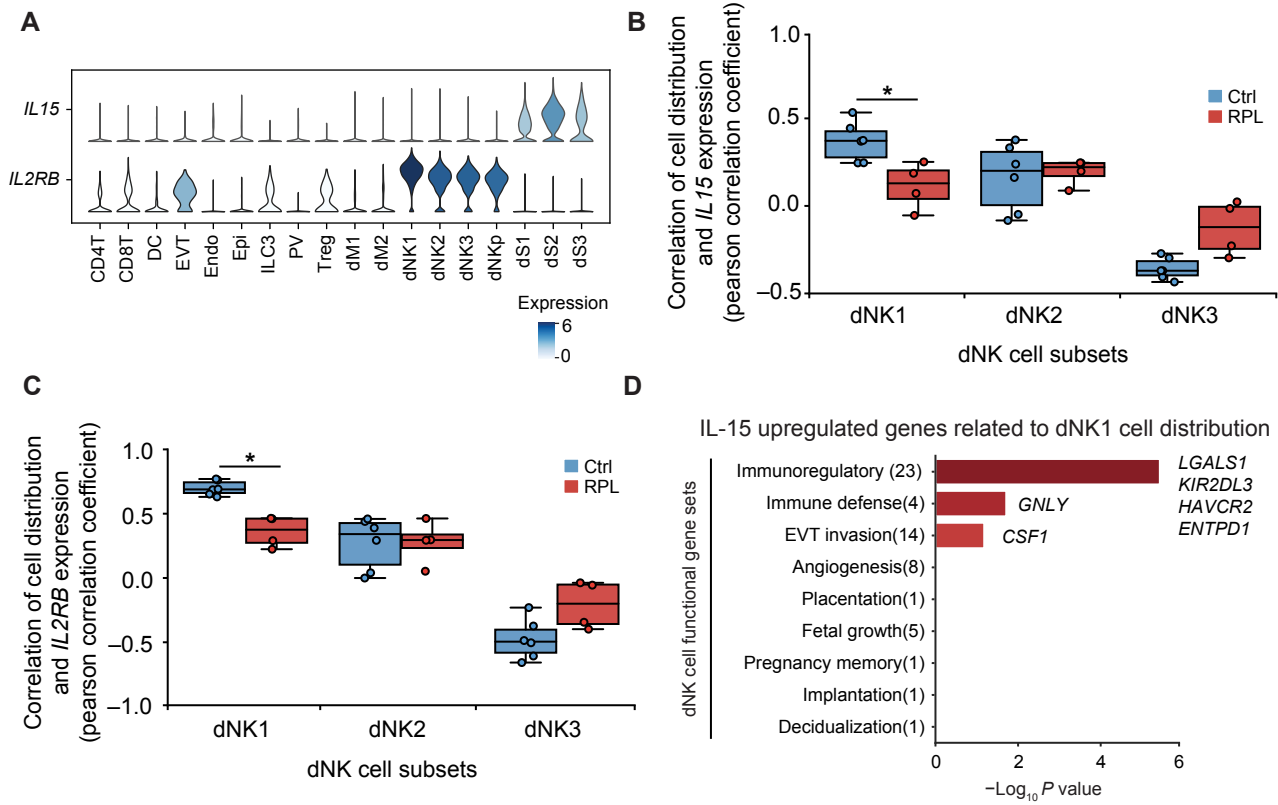

Supplement: qzaf080_Supplementary_Data [file qzaf080_supplementary_data.zip › Figure S6.pdf]

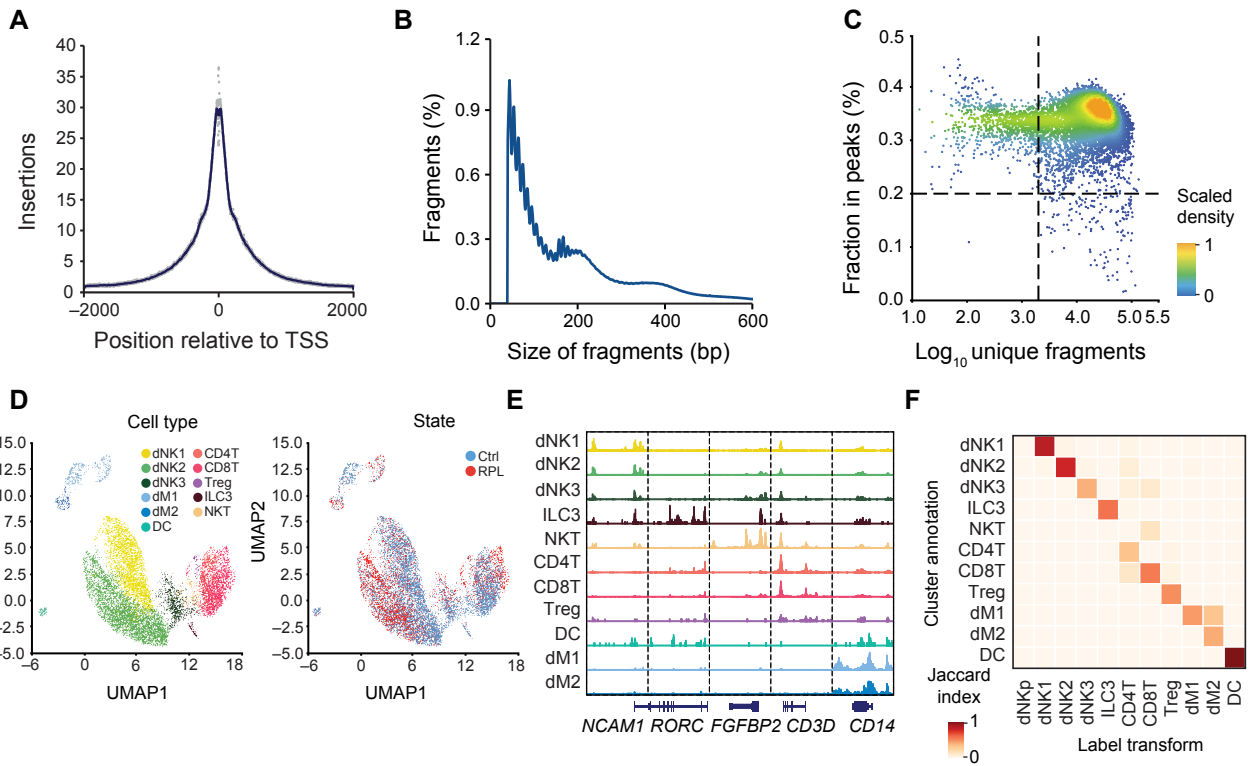

Supplement: qzaf080_Supplementary_Data [file qzaf080_supplementary_data.zip › Figure S7.pdf]

**A**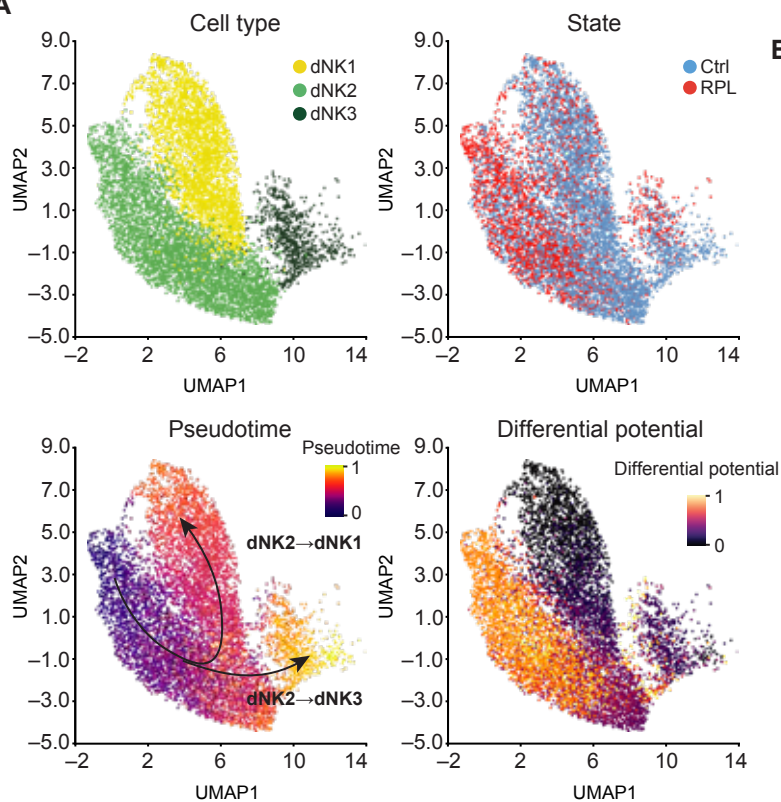**B**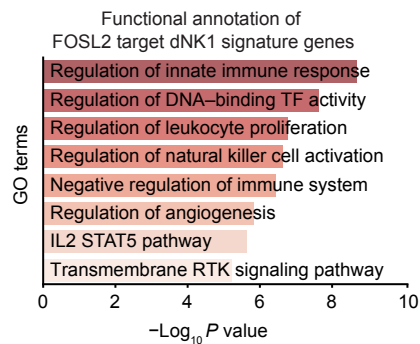

Supplement: qzaf080_Supplementary_Data [file qzaf080_supplementary_data.zip › Figure S8.pdf]

**A**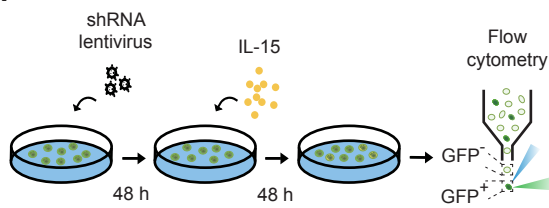**B**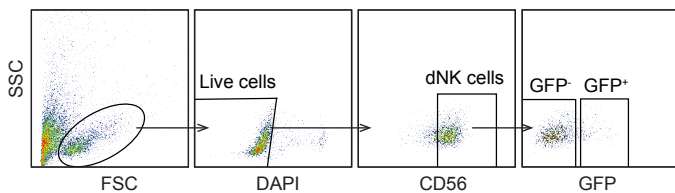**C**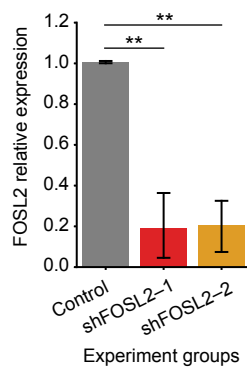**D**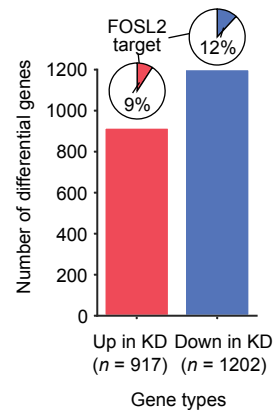**E**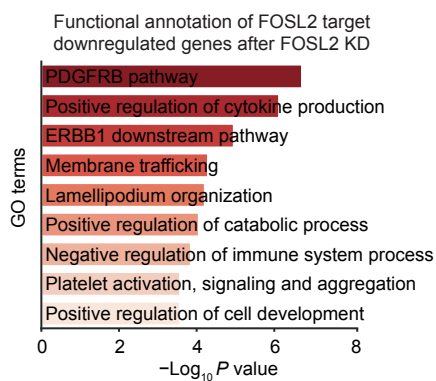**F**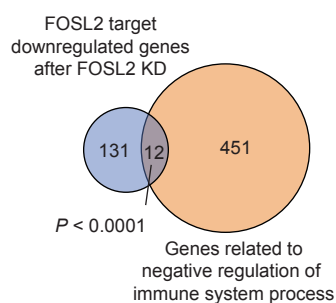**G**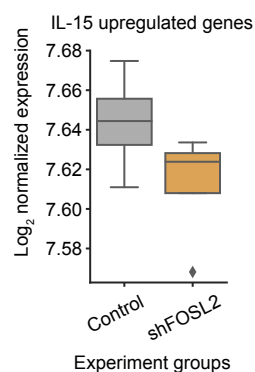

Supplement: qzaf080_Supplementary_Data [file qzaf080_supplementary_data.zip › Figure S9.pdf]
